# Rhythmic subvocalization: An eye-tracking study on silent poetry reading

**DOI:** 10.16910/jemr.13.3.5

**Published:** 2021-09-14

**Authors:** Judith Beck, Lars Konieczny

**Affiliations:** Cognitive Science, University of Freiburg,, Germany

**Keywords:** Eye movements, poetry, silent reading, rhythm, subvocalization, auditive expectation, meter, rhyme, load contribution

## Abstract

The present study investigates effects of conventionally metered and rhymed poetry on eyemovements in silent reading. Readers saw MRRL poems (i.e., metrically regular, rhymed language) in two layouts. In poem layout, verse endings coincided with line breaks. In prose layout verse endings could be mid-line. We also added metrical and rhyme anomalies. We hypothesized that silently reading MRRL results in building up auditive expectations that are based on a rhythmic “audible gestalt” and propose that rhythmicity is generated through subvocalization. Our results revealed that readers were sensitive to rhythmic-gestalt-anomalies but showed differential effects in poem and prose layouts. Metrical anomalies in particular resulted in robust reading disruptions across a variety of eye-movement measures in the poem layout and caused re-reading of the local context. Rhyme anomalies elicited stronger effects in prose layout and resulted in systematic re-reading of pre-rhymes. The presence or absence of rhythmic-gestalt-anomalies, as well as the layout manipulation, also affected reading in general. Effects of syllable number indicated a high degree of subvocalization. The overall pattern of results suggests that eye-movements reflect, and are closely aligned with, the rhythmic subvocalization of MRRL.

This study introduces a two-stage approach to the analysis of long MRRL stimuli and contributes to the discussion of how the processing of rhythm in music and speech may overlap.

## Introduction

*To recapitulate then:*

*I would define, in brief,*

*the Poetry of words as*

*The Rhythmical Creation*

*of Beauty.*

*Edgar Allan Poe, The Poetic Principle*

If you know Grimm’s fairy tale Rumpelstiltskin, you might agree that these lines are funny: *Ha! glad am I that no one knew, | that rhythmic guitar I do play well.*

Why is that? The original – *“Ha! glad am I that no one knew | That Rumpelstiltskin I am styled”* (48) – has a strict metrical structure. This creates a regular rhythmic pattern, whereas in the introductory example, the stress pattern deviates from the expected metrical scheme. It is difficult to accommodate to that deviation rhythmically. Thus, it prompts an experience of rhythmical oddness in the second line (which you might have just experienced while reading silently). That, in turn, justifies the sentence’s content, i.e., why it might be good that nobody knows that the speaker plays rhythm guitar, as he or she appears to be lacking talent.

While establishing speech rhythm is not at all trivial, rhythmic patterns appear to be a relatively easy cognitive task in regularly metered and rhymed poems even for children (118). However, it remains unclear how it works in silent reading MRRL (i.e., metrically-regular, rhymed language), if *subvocalization* plays an important role in it, and whether eye-movements may reflect that.

### Subvocalization and eye-movements

*Subvocalization* serves to ‘prepare for pronunciation’, but is simultaneously characterized by inhibited speech-motor articulation (84, p. 2). It may involve hearing an *inner voice* (1; 27; 59; 107 ). Fluent silent reading is usually preceded by the different stages of learning to read orally (91), supposedly with varying degrees of subvocalization. Thereby phonological awareness is crucial for expressing prosody as well as coordinating temporal predictions for speech rhythm (92). When reading silently, a reader’s inner voice can also distinguish inertly between various qualities, mirroring external intonation and modulation, varying i.a., by volume/stress, pitch and tempo (142). Hence, subvocalization can affect silent reading (79; 131), which is reflected in the implicit prosody hypothesis. It states that phonological features influence syntactic parsing and guide ambiguity resolution (8; 42, 43, 44). For example, gaze durations and fixations are modulated by the number of stressed syllables within a word (6). Also, syntactic analysis is affected by the *alternating* distribution of stressed and unstressed syllables (63, 64; 65) and stress perception can be influenced by suprasegmental cues such as the preceding stress distribution (21). Importantly, in experiments using Limericks, syntactic reanalysis of a critical region elicits longer reading times when it requires a reanalysis of the metrical pattern (18, 19). This supports the notion of stress expectation management (120, 121). These findings indicate that (lexical) stress registered in eye movements is based on phonological mental representations and that “readers form an implicit metrical representation of a text during silent reading” (19, p. 1896). ERP results presented by Breen et al. (20) offer first evidence that explicit and implicit metric may be processed similarly.

However, to date, the nature of this representation is not yet well understood (16). Is it abstract in the sense that it is a-modal, i.e., stripped of any sensory-motoric representations, and does it require at least some representation of sound, or even the - yet suppressed - execution of motor-action? There is initial evidence that eye movements during silent reading are influenced by subvocalization (29) to the point that the spatial distance between the eyes (that lead) and the voice (that follows) might affect and even regulate eye movements (84), even in silent reading. With this in mind, subvocalization should play a key role in silent reading for the adaption of a MRRL-text’s metrical figures and rhythmic contour. The following questions arise: 1. Would an MRRL-rhythm, bearing a ‘purposeful’ audible ‘gestalt’, be perceivable to readers reading *silently*? 2. If so, would eye movements show sensitivity to anomalies within a rhythmic ‘gestalt’? 3. Can we observe eye-movements suggesting *subvocalization* of a rhythmic gestalt and if so, in which measures (113; 114)?

### Metrically regular, rhymed language (MRRL)

Poetry, with *traditional* meter and rhyme, is considered melodic, being both music *and* language (94). Historically, oral traditions preceded written compositions. Furthermore, a major aspect is rhythmicity. For Poe, the ‘rhythmical creation’ is essential for the ‘poetry of words’, i.e., a poem’s rhythm is caused by words' respective sounds appearing in a specific order, by which they build an audibly perceivable ‘gestalt’ (23; 70; 86; 87; 98; 99; 129 ; 139). In silent reading MRRL, this ‘gestalt’ would then have to be instantiated by the reader.

a) *Meter* is a contributing factor to an audible gestalt (35) in MRRL. In oral reading, its hierarchical nature is realized via two distinct means, intensity (39 ) and duration (17). Traditionally, it relates to the percept of an alternation of stressed/accented (strong) vs. unstressed/unaccented (weak) syllables (103; 109 ; 126). Meter is proposed to influence cognitive fluency, memory and verbatim recall (compare 3; 102 ; 135; 141), and to aid temporal-based predictive language processing and comprehension (30 ; 95; 117).

We define meter according to Ravignani & Madison (112) as the “hierarchical organization of temporal events based on stress and other spectral properties, such as loudness alternation, pitch variation, etc.” While *silently* reading MRRL, hierarchically, temporally, and spectrally shaped stress patterns may be represented, inferred and automatically categorized into metrical entities (i.e. a (linguistic) metrical grid, see 86, p. 5). An abstraction of their overall sonic similarity distribution is then projected onto what is expected in the next line or stanza, altering the “mode of attention” (47).

The frequent and structured repetition of a set of metrical figures contributes to their prominence and perception as regular. This, in turn, allows for beat extraction and induction (55, 57). Here, beat is “psychologically superimposed” and can be defined as the “isochronic grid generated via metrical expectations” (111 ; 112, p. 2). This grid is marked by a “rhythmic pattern, where all intervals have *roughly* equal duration” (ibid., authors emphasis), whereby rhythm can be understood as a durational-bound pattern of events within a time-frame (ibid., see also 88; 123; 143 ; 147). Due to its phonological rhythmicity, durational pattering, and structural repetition of meter *MRRL* supposedly offers a higher level of ‘isochronicity’ than normal speech (for an investigation of stable periodicity see 112).

Although an inferred beat may inwardly ‘go on’ autonomously while reading, the respective prominent metrical figure has to be checked and updated in order to maintain it. Therefore, it must be aligned constantly with the upcoming input (for normal speech see 10 ). This process is based on two levels: a) downright processing of local stress grids as required by the phonemic-syllabic material (87, p. 261) and b) actualizing the underlying (physically or non-physically salient) quasi-isochronic MRRL-beat. However, both, rhythm and meter can change from one stanza to another or even from one verse to another. In this case, readers must attune their temporal predictions, either by inferring, respectively projecting a new ‘metrical grid’ to the following lines, or by adjusting to an accelerated/slowed-down beat, i.e., applying slightly increased or decreased intervals (112). If reading MRRL silently not only demands beat extraction but also requires successive beat induction (56) it should, in turn, create tension, and, if an expectation is not fulfilled, a sense of violation.

Possible changes to a rhythmic structure can be a violation of the number of (inferred) beats, a deviation by one syllable less or more, a substitution of a word by another one which demands preponed or delayed accent/stressing (see 4 for rhythmic tone sequences), or a new “sound” (vowel) that changes a gesture. Therefore, characteristics of phonemes, such as tonal weight/sonority, duration level, loudness, breathiness, e.g. /s/ vs. /a/, are likely to contribute to recognition and processing in silent MRRL reading at a very early stage, as it does in oral speech (122; 152 ). These contrastive and coordinative features (100 ) as well as their related articulatory, co-articulatory and accentual gesture qualities (136), creating i.a. phenomena such as sound diffraction or floating stress, are at play in the consecutive order of syllables/words. Without this order, there would be stress but no ‘regular meter’, no ‘beat’ – and no *structured* MRRL-rhythm. Therefore, we propose that for MRRL, like for music, “meter involves *when* events will happen, while grouping involves *what* events will happen” (90, p. 6). This notion is based on the assumption that for MRRL, “the strongest correspondences between music and language appear to be between musical syntax and linguistic phonology, not musical syntax and linguistic syntax” (87, p. 257).

b) *Rhyme* as a stylistic device is the second important factor shaping the ‘audible gestalt’. It contributes to a text’s coordinating auditive characteristics by structuring the stream of words, respectively syllables, via repetition (33) and via sonic modification, e.g. perfect vs. imperfect rhymes (69 ; 124, p. 350), such as ‘blind/mind’ vs. ‘line/find’. Readers or listeners of a poem seem to be sensitive towards rhyme schemes (22; 102). Scheepers et al. (119), for instance, found strong effects of rhyme anomalies in listeners’ pupillary responses. Hence, in MRRL, the formation of expectations of what is to be ‘heard’ or ‘seen’ in the next line is also triggered by the circulation of end rhymes as part of a larger time scale (31) or internal rhymes (60 ; 62), supposedly related to smaller time scales. Importantly, as suggested by Schrott & Jacobs (124, p. 352), the verse-end position of rhyme is crucial for determining the meter of a line, and at the same time divides a poem into segments. The poem’s line as the salient and fundamental structural unit of verse marks boundaries for readers, in conventional poetry mostly via end-rhymes (34 ; 36; but see 53 for the genre of prose poetry).

These boundaries often elicit pausing, supposedly due to closure effects (130) and enhanced by the poem’s visual presentation. Naturally, pauses are crucial, too, for the production and detection of MRRL-rhythm. They have an explicit attentional function for maintaining rhythm (46) and for directing breathing patterns in oral reciting, bearing the potential to be mapped onto subvocalized reading patterns. Turner & Pöppel (140) proposed a time unit per verse (2-4s, average peak around 2.5-3.5s), which may shape reading/reciting MRRL and contribute to temporal prediction and segmenting, regardless of number of syllables (for a critique of the 3-sec-postulation see 32 ; but see 66 for a comparison of durations of lines with biological action units; 145, 144 ; for a review see 153). Ultimately, however, the overall regularity which allows for MRRL-rhythm because of stylistic devices, such as meter and rhyme or other parallelistic dictions (for details see 95; and 93 ), is dependent on the phonological material of a poem (67).

c) *Layout*. Most of the ongoing discussion about the role of layout, i.e., poem vs. prose (31; 49), as well as the function of features such as rhyme, focuses on the potential to affect categorization, reading strategy and tempo, comprehension and memory processes as well as aesthetic appreciation (50, 51 ; 54; 96; 108; 148; 155). Important in the context of our study is that when reading poetry, top-down processes, termed genre-effect, can impact attention strategies (49, 51). Furthermore, eye-movement patterns can differ when the same text is presented in poetry or in prose layout. Fechino et al. (36) found overall longer gaze durations and a higher rereading probability in the poetry layout. Our present study has a similar design but different focus, and was completed and submitted before Fechino et al.’s study was published. The same holds for findings on the processing of rhyme and meter (96). Amongst other results, they report ‘total gaze durations’ to be longer for verse-final words, when either rhyme or meter or both were present, and findings were interpreted within the aesthetic emotions approach (but see 128).

### Entrainment and MRRL

As stated earlier, to perceive a rhythm or to induce a beat, we must be able to synchronize and/or to entrain to a stimulus (55). Importantly, the term entrainment originally refers to *external* stimuli provoking an internal pattern of neuronal responses that seem to be rhythmically aligned with and periodically reflect or represent (external) stimuli, such as light (41, p. 48), sound (452), rhythmic auditory stimuli (101) or music (132, 133 ). With speech, processing appears to be bound to timing patterns, too, for the auditive input as well as for the responding neuronal activation (154). Kotz et al. (75, p. 896) propose that “we seem to neurally synchronize with rhythm in speech, which captures our attention, regularizes speech flow, [and] may emphasize meaning” (77). In silently reading MRRL, perceived meter and rhythm may affect neurocognitive oscillators (109) and may elicit a perception of periodicity (75 ), even in the absence of an explicit signal. This, in turn, may lead to synchronization with an isochronal pulse (but, in terms of music, may not, see 89). Further support comes from the fact that production and processing of music and language share neuronal circuits (37; 80; 106; 115). As entrainment to music goes along with beat induction (55), we presume that beat extraction and induction works for MRRL, too, and may be a theoretical basis for the explanation of the cognitive phenomenon of rhythm effects (104, p. 913).

### Aim and rationale of the study

To our knowledge, up until now no one has investigated the role of subvocalization linked to rhythm in silent reading of MRRL-poetry. Here, we propose that MRRL serves as an acoustic stimulus inwardly brought to mind via rhythmic subvocalization. As such, it is bound to timing and bears the potential to be entrained (28; 74 ; 75, p. 902; 76; 97 ; 133). Accordingly, we hypothesize that readers pick up MRRL-rhythm when they read with an inner voice and, thus, that they should experience a sense of violation if the accuracy and predictability of MRRL is interrupted. The question is if and how this is reflected in eye movements.

Beyond phonological properties of MRRL, we were interested in the extent to which the line layout contributes to the rhythmic perception of MRRL-poems. If line breaks are used as additional rhythmic cues, it should, on the one hand, be more difficult to pick up the metrical grid and rhythm structure when poems are presented in prose form, i.e., when verse endings do not always coincide with actual line breaks. On the other hand, because the rhythmic and audible ‘gestalt’ of MRRL must be updated constantly, we suspect that reading is influenced by a text’s (poem/stanza) sonic cues (compare 5; for a general discussion of the importance of phonologicy see 12). Hence, MRRL rhythm should also be picked up in the prose layout, albeit leading to different eye movements compared with the poem layout.

So, firstly, we were interested in whether readers would take on an MRRL-rhythm at all. To test this, we introduced three types of anomalies at significant places in the poems: *metric* anomaly, *rhyme* anomaly, and a *combination* of both. A metric anomaly is a deviation of the expected linguistic metrical grid, at a specific location. This grid should govern the subvocalization of the line/stanza until the rhythmic inconsistency has to be processed. Salient deviations should result in a noticeable slowing-down in reading if they are experienced as ‘violations’ (compare 19), which would imply that the MRRL-rhythm had been picked up. For rhyme anomalies, Scheepers et al. (119) report stronger reactions in pupil dilation than for metric or other anomalies. Thus, we also would expect rhyme anomalies to elicit longer reading times. For combined rhyme and meter anomalies, the single effects for rhyme and meter could, on the one hand, add up and thus lead to the longest reading times for this anomaly type. Also, the combined anomaly might impede the accommodation of the rhyme scheme. However, on the other hand, the combination could lead to the disintegration of the rhythmic structure, i.e., this anomaly might not be experienced as an expectation violation at all.

We expected the type of anomaly to interact with the line-layout of the poem. In the *poem* layout, the original verse-structure is preserved, whereas in the *prose* layout, line breaks, most of the time, do not coincide with verse endings. In the *poem* layout, the rhyme structure is clearly identifiable, as the end of verses coincide with line endings, whereas the rhyme words are hidden somewhere within the lines in the *prose* layout. This might have two consequences: First, it should be harder to pick up the rhyme scheme in the *prose* layout, and hence divergences from the given rhyme scheme might go unnoticed. Secondly, if a rhyme anomaly is detected, the pattern of re-fixations might differ, as the first word of a rhyme pair – called pre-rhyme (130) throughout the rest of the paper – is harder to detect in the prose layout, as its position is presumably more difficult to memorize.

The layout might also affect the processing of metric anomalies, because their detection might be easier in layouts with a strict verse-by-verse structure typical for poems.

Furthermore, we also expected re-fixations to the origin of the anomalies where possible. In rhyme anomalies, the origin is the corresponding rhyme word usually at the end of a verse above, whereas meter has no such clear origin, as it is construed across entire verses. However, since the units of rhythmic gestalt are comprised of only a few syllables, the immediate context of a metric violation is much more important than for rhyme anomalies. This should result in more local re-fixation patterns for metric violations, regardless of layout. Rhyme anomalies are expected to elicit more across-line refixation on the pre-rhyme.

**Figure 1.**
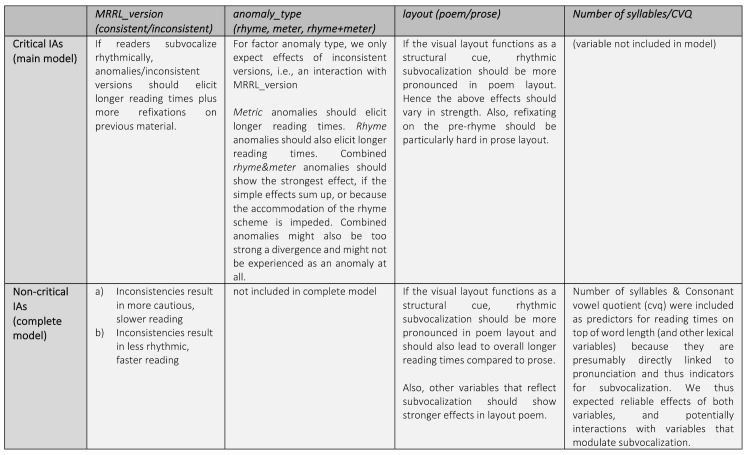
Illustration of hypotheses for main and complete model

On a more general level, we were also interested in identifying indicators of MRRL-triggered subvocalization in our eye-tracking parameters throughout entire poems. In particular, we were interested in how the introduction of anomalies and the layout versions would modulate reading in general, not only at critical interest areas (see *figure 1*).

Note that we have not included obvious semantic or syntactic anomalies in this study. Poems (or poetic language more generally) may induce a certain tolerance towards these kinds of violations (see^13^ for investigation of genre-related tolerance towards semantic and morphological anomalies in verse, and syntactic inversions, 14), but this is not a research question of this paper.

## Methods

### Participants

Thirty-eight participants (23 females; 15 males, mean age: 28.87 years; SD age = 12.33 range: 19-76 years) took part in the study. They were recruited via the Sona System within the Department of Psychology, University of Freiburg, Germany, via notices on bulletin boards at different faculties and via email to distributors like art associations, the House of Literature Freiburg or the Freiburg University of Music. All participants were native speakers of German. 29 were students or participants without clear indication of profession/status (average age: 24.9), 8 were employees (average age: 37.38), 1 was retired (aged 76). All of them had normal or corrected-to-normal vision and were naïve to the experiment’s purpose. Subjects received either course credit for participating or alternatively signed up for a lottery drawing with the chance to win 3x 45 min of scientific or creative writing training.

### Ethical Statement

No invasive or unsafe methods were applied and only behavioral data such as eye tracking data and questionnaires were collected. All participants gave written consent before the experiment started. The experiment was conducted in accordance with the standards of the “Ethical Principles for Medical Research Involving Human Subjects” (Declaration of Helsinki, 1964), set by the World Medical Association. This study was conducted according to the DFG-guidelines for good scientific practice, including originality of research idea, experimental design and method used, and is devoted to fair research behavior.

### Apparatus

The experiment was designed and the study was conducted in spring/summer 2019 in the Cognitive Science eye-tracking laboratory at the *Center for Cognitive Science*, University of Freiburg. The reading experiment was set up with the ‘Experiment builder’ software (SR Research Ltd., Mississauga, Canada). Using the SR Research EyeLink 1000 (SR Research Ltd.) eye-tracking system, participants’ eye movements were recorded, with a sampling rate of 500 Hz and an accuracy of 0.25° to 0.5° of the visual field. To reduce head and body movements, a chin-and-head rest was securely mounted on a table. The distance between the EyeLink 1000 chin-and-head rest and the screen was 60 cm. Only the right eye was tracked. Before eye-movement recording was started, standard 9-point calibration and validation procedure was executed to gain a spatial resolution error of less than 0.5° of the visual angle.

### Design and Materials

Stimuli consist of eight German poems that were each manipulated according to a 2x2 design, comprising the factors *layout* (poem vs. prose) and *version* (original poems vs. versions that included rhythm and rhyme violations). The 8x4 (items x condition) texts were then distributed to four presentation lists following a Latin square rotation scheme, such that each participant was presented with two texts for each condition, and each item occurred only once per list.

The order of presentation of stimuli was randomized. Stimuli were presented in Trebuchet MS, with a font size of 30. The display resolution was 1920 (width) x 1080 (height) pixels, leaving space for up to 13 lines of text with a 1.5 line spacing. Stimuli were split over max. 3 pages of the screen (for poem versions: page one presented stanza 1-3, page two stanza 3-6, page 3 stanza 7; for prose versions: page 1 presented the first two text blocks, consisting of stanza 1-4 and page 2 presented the second two text blocks consisting of stanza 5-7).

Although the prose version caused one critical region to coincide with the position of the last word on the screen, which is commonly known to be a problematic area regarding eye-movement behavior, we decided to keep this structure to examine effects caused by a disruption of expected rhythm at the end of the rhythmic system (auditive gestalt) of the prose version, as well as the poem version. This decision was also based on results reported by Wassiliwizky et al. (146), who measured skin conductance to investigate emotion and aesthetic appreciation while listening to poems and found that chills occurred at the end of line, end of stanza and end of a poem.

**Figure 2.**
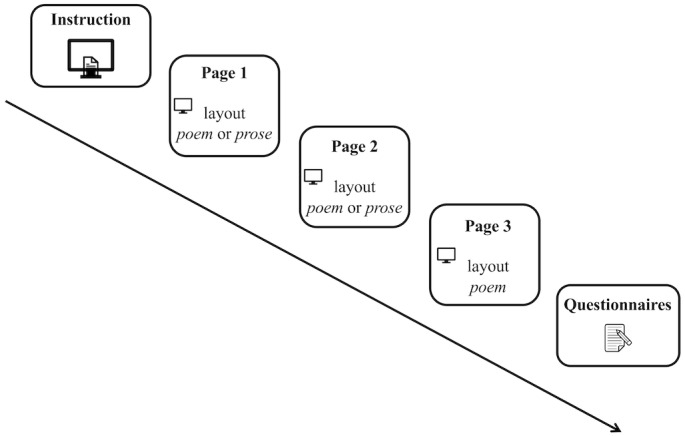
Illustration of experimental setup.

**Figure 3.**
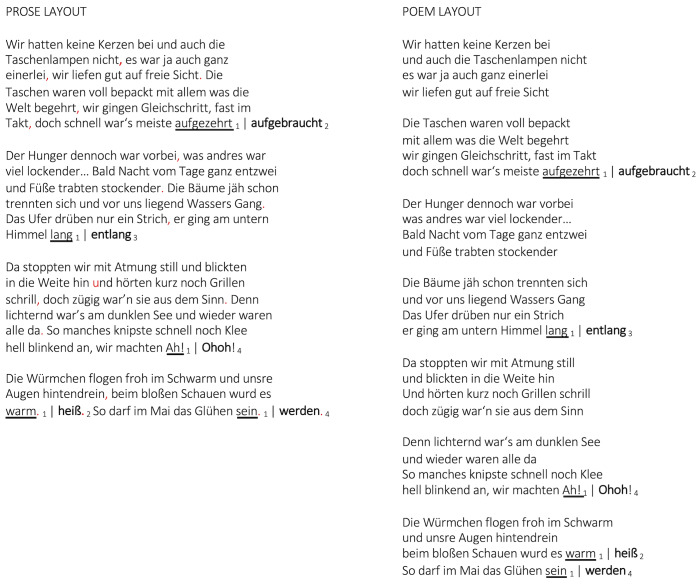
Illustration of poem layout and prose layout: (1) original text, (2) *rhyme anomaly*: substitution of rhyme with original number of syllables, (3) *metric anomaly*: change of prominent metrical figure by adding one or two syllables with rhyme being maintained, (4) *rhyme and metric (rm) anomaly*: change of prominent metrical figure by adding one or two syllables, with substitution of rhyme.

The three types of experimental manipulations (*meter rhyme*, *rhyme&meter*; see appendix for all stimuli) are shown *figure 3*.

The first stanza of a poem introduced its rhythm, so participants had the chance to pick it up while reading silently and to potentially build rhythmic expectations. The rhythm of each poem was closely aligned to its main metrical grid to make sure MRRL was strongly metrical (compare figure 2 in 112), thus allowing for ‘quasi-isochrony’. We also added combined rhyme and metric anomalies (manipulation 4). These anomalies presumably impede the accommodation of the rhyme scheme into an ABAC pattern.

Stanzas 3 and 5 were in accordance with the rhythmic constraints so that readers might pick up the rhythm again. Manipulations (2) and (4) in stanza 7 allowed for complete deviation from the ABAB rhyme-scheme. In the present study, ABAB scheme implies perfect as well as imperfect, but acoustically close rhymes. Findings by (69, 10f) suggest “that imperfect rhymes benefit from metered verse context” and “are harder to distinguish from perfect rhymes as distances increase”, presumably depending on the “degree of phonological similarity”.

Note that we introduced the different rhythmic deviations on the basis of the constraints named above (for details see Appendix).

*Rhyme anomaly.* Since the first stanza introduces the ABAB scheme, readers may use it as a default for the upcoming stanzas. Rhyme anomalies such as *begehrt*/*aufgebraucht* instead of *begehrt*/*aufgezehrt* do not violate a potentially superimposed regular beat distribution but they may collide with the expected rhyme scheme. This holds true for imperfect rhymes, too.

*Metric anomalies* were construed by adding one to two syllables, disturbing the grouping structure of the previous syllabic material in the stanza. This was done by e.g., violation of expected stress/accent, by missing and/or delayed accent or by preponed and/or added accent. Examples are e.g., *Gang*/*lang* vs. *Gang*/*entlang*, leading to an additional floating stress moment, or *grad*/*Waldesnaht* vs. *grad*/*Waldesziernaht*, leading to preponed stressing of “zier” and stress diffraction on the last syllable “naht”. Adding a syllable could also shift the projected number of beats if introducing one more syllable which requires stress, thus locally disturbing the overall stress distribution within the stanza.

*Metric & rhyme anomaly* should most clearly lead to irritation within the overall rhythmically structured ‘gestalt’, either by realizing possibilities listed above combined with deviation from the rhyme scheme, or, by implying a stress clash, e.g. gegeben/(gut) durchleben vs. gegeben/(gut) überstehen (see Appendix for further details). However, our focus was not to analyze the different sub-types of metrical anomalies or rhyme anomalies, but more so the general eye-movement reactions elicit by the anomalies.

The corresponding prose version which includes experimental manipulations had the same pattern (adjusted interpunction marked red). Content-wise, both prose versions, original and manipulated, were in line with the corresponding poem versions. Changes in prose versions were undertaken for two purposes: a) line breaks should not coincide with the position of pre-rhymes, and b), when line breaks coincided with clause boundaries in the poem layout, interpunction and capitalization was adjusted to preserve the clause structure.

Seven poems were composed by the first author specifically for the purpose of the experiment. One more poem was an original, “Auf hohem Gerüste” (116, p. 63; excluded from data analysis). Hence, the stimuli have not been used in previous research. These seven poems followed a preset poetic rhythm structure as close as possible. This was obtained by adherence to the rhythmical matrix of classical originals, i.e., 1) *Dancing Queen*, 2) *Flüstern*, as in “Der Pilgrim” by Friedrich Schiller, 3) *Klimawandel* as in “Der Wanderer in der Sägemühle” by Justinus Kerner, 4) *9 Leben* as in “Auf hohem Gerüste” by Joachim Ringelnatz, 5) *Normal* as in “Am Waldessaume träumt die Föhre” by Theodor Fontane, 6) *Im Hüteland* and 7) *Glühwürmchen* were authored following preponderantly the rhythmic matrix (rhyme, meter, phonological relatedness) of those named above. They all had to rhyme according to the ABAB-scheme, which could also include imperfect, yet acoustically close rhymes.

The semantic field of words was chosen from commonly known topics such as nature, summer, youth, desperation, etc. Poems mostly contained familiar and high frequency words, such as *luck, stars, sky, forest, breathing*, etc., function words as well as some low frequency or antiquated words, and neologisms.

The seven new poems included parallistic dictions and a higher level of difficulty (26; 150, 151) compared to “Auf hohem Gerüste” by Ringelnatz, i.e., they presented a moderate number of stylistic devices such as assonances, alliterations, comparisons, e. g. “die Sterne wie Glitzerstuck am Himmel” (the stars like glittering stucco in the sky) or neologisms, e.g. “Hügelzwerg” (hill dwarf), etc. We did not exclude any non-standard syntactic patterns, because word order is an important stylistic feature contributing to the multi-layered meaning and rhythm construct of a MRRL-poem (124).

Although stimuli were written in a sound-familiar metrically regular and rhymed style (such as quatrains, nursery rhymes, etc.), the choice of words and the occasionally complicated syntax should prohibit complete and deep sentence comprehension. At the same time, we expected readers to grasp the narrative of a poem quickly (25), i.e., global comprehension of content. For this reason, we assumed fluent reading, which in turn was presumed to enhance rhythmic subvocalization. Also, participants were not allowed to move back to earlier pages, which also made full sentence comprehension within the course of a poem more difficult. This ensured that rhythm became a more salient feature.

### Procedure

The experiment was designed and conducted in the Cognitive Science eye-tracking laboratory at the University of Freiburg. Participants were asked to sit at a desk across from the eye-tracker table, to read a brief information sheet and to give written informed consent prior to the experiment. Next, they were asked to sit in front of the eye-tracker. Body position adjustment and camera setup (calibration and validation) were undertaken.

The recording session started with a short instructional text on the screen (see appendix for exact wording). Its purpose was to acquaint participants with reading in front of an eye-tracker with their head in a head-and-chin rest. The text informed participants about the fixation cross and the space bar so that they would know how to proceed to the following page. It also invited them to be curious about the content and asked for their attention to the upcoming texts. No instruction for reading speed was given. Stimuli were presented in randomized order for each participant. At the beginning of each trial participants had to fixate on a cross at the position where the first word of the item would appear and press the space key. Once they did so, the first page appeared on the screen. When they finished reading a page, participants could move to the next page by again pressing the spacebar. No option for moving back to the previous page was provided. After they had finished reading the last page of a trial, the next trial was indicated by the next fixation cross.

After the recording session was finished, participants were asked to sit again at the first desk and to fill out questionnaires (1. processing of stimuli, 2. reading habits, 3. *A short Questionnaire to Assess Musical Activity*, MusA (38 )). Participants were allowed to ask questions with regard to proper understanding of questions (such as: “Does this question apply to all texts?”). Answers were only given when necessary. Otherwise, participants were invited to read again closely and to give an answer that would seem appropriate to them.

After finishing all three questionnaires, a short feedback interview took place with questions like “Did you notice anything special about the texts?”. If key words like *rhythm*, *expectation*, *(inner) voice* or semantically or thematically close words were part of an answer, participants were asked to specify what they meant by these words. In addition, they were asked whether they had noticed something about their eyes or whether they had inwardly heard something like a voice during reading silently or not. If the latter was confirmed, they were asked to try to explain a possible function of that inner (reading) voice. Notes of answers were jotted down. Questionnaire and interview data has not been included in the analysis and will be discussed elsewhere.

### Data Analysis

Fixation reports of the raw data were generated using the SR-Research Data-Viewer. Blink durations were not included in fixation durations. Fixations occurring directly before or after a blink were not excluded from the data set. Rectangular interest areas (IA) were defined automatically around each word on a page. Every computational step from here, including interest area assignment, was taken in the R programming language. The code is available upon email request.

For each fixation, we assigned an IA based on the fixation’s x and y coordinates. Fixations’ start times were used to identify the page - one out of three - that was read. The completed fixation reports were then transformed into IA-reports, with each row representing a consecutive IA/word in an item, including variables for eye tracking measures, lexical features and other IA related variables that would potentially affect reading measures, including the design factors.

Word reading time measures, especially in longer texts, are affected by many variables that are not in the main focus of our study. However, to control for these variables, we consider it mandatory to account for their influence. This should be done on as many data as possible, namely on all words in the texts, with the exception of the first word.

For the data analysis of the critical IAs, we hence chose a two-stage approach, where the analysis of critical IAs was based on residuals derived from all IAs.

**Figure 4.**
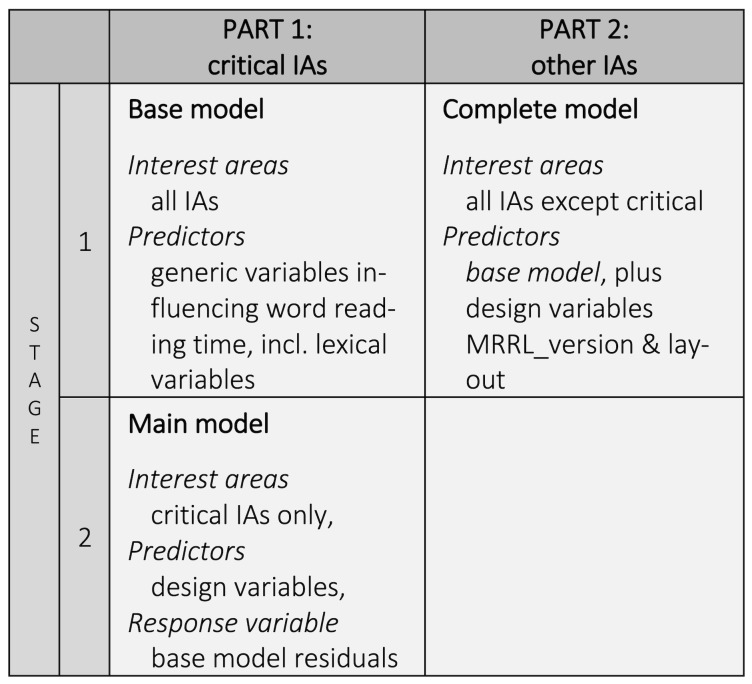
Scheme of analysis.

However, we were also interested in general eye-tracking signatures of subvocalization on areas other than the critical IAs. We therefore chose to analyze the IA-reports in two parts (except for skipping probability and load-contributions; see *figure 4*).

Part 1 focused on the reading of the critical IAs themselves. This analysis has been carried out in two stages. In stage 1 we fitted a base model over all IAs (words). The purpose of the base model is to eliminate all effects that are not (related to) the design factors, which are included in the main model, namely *layout, anomaly_type* and *MRRL_version*. The base model includes a wide variety of general predictors that are known or very likely to influence eye-movements and word reading times. Among those were *i.* lexical features, such as word length, frequency (61; 68; 125 ), and the word category (noun, verb, adjective, closed class words), *ii.* structural features, such as whether an interest area (word) occurred at the end or the beginning of a line (73) or verse (rhyme indicator) (22 ). Finally, we also included *iii.* oculomotor behavior variables, such as whether or not a first pass regression is launched, and gaze durations of the predecessor word. These variables can strongly affect all duration measures independently of our design factor manipulations and should thus be accounted for, either in the base or main model. Accounting for them in the base model has the advantage of almost completely detaching them from the critical IAs, where the effect of the design variables should be as pure as possible.

*The base model* was only fitted to produce residuals (138), which were then used as the response variables in the second stage models. Using residual reading times is a common technique to account for, and eliminate, irrelevant influences before looking at the effects of the design factors. Note that the base-model was fitted across *all* interest areas.

The residuals were then used in stage 2 (i.e., the main model) to analyze a reduced data set, where all but the critical interest areas (IAs) were excluded. Because distractor influences were eliminated in stage 1, the main model only included the design factors as fixed effects predictors. Critical interest areas were those target words that have been manipulated in the experimental conditions, i.e., replaced with other words inducing a meter or rhyme anomaly, or both.

The two-stage approach was chosen for two reasons: First, we could include a plethora of variables influencing reading times in the base model without sacrificing power in the main model. The main model could thus be based on residual eye-tracking parameter values that were fitted over the entirety of the poems, consisting of about 160 words each. Had we chosen to include all predictors in a single model, not only would we have lost power by analyzing only five interest areas (words) per poem. Secondly, estimates of lexical variables would have been obscured by any manipulation that disrupts reading, particularly so the anomalies. Only results from the main model of part 1 will be reported.

*Complete model.* However, we were also interested in how our design manipulations affected reading in general - not only at the target words, but throughout the entire poem. Hence, part 2 focused on the effects of our manipulations on all but the critical IAs. This *complete model* included all predictors from the base model in stage 1, plus the design factors *layout* (layout: poem vs. prose) and *MRRL_version* (consistent vs. inconsistent). Factor *anomaly_type* was not included, because it was only defined for critical IAs, as all manipulated stimuli contained all three types of anomalies (anomaly_type metric, anomaly_type rhyme, anomaly_type r&m).

For all analyses, *linear or logistic mixed effect regression* models were fitted using the *lme4-*package (7; 9) in R (110 ). Further packages used were *LMERConvenience Funcions* (137), *lmerTest* (81), and *multcomp* (58).

For the complete models, we used stepwise elimination to yield a minimal model, which only included predictors that significantly increase the model quality. For this, we used the function step() from the *lmerTest* package, which applies backward elimination of random-effect terms followed by backward elimination of fixed-effect terms in linear mixed models.

The variance inflation factors of all predictors in both the main and complete models were below 5.

*The main model* of part 1 included the study design factors *layout* (*layout* with levels *poem* vs. *prose*), *MRRL_version* (with levels *inconsistent* vs. *consistent*), and *anomaly_type* (levels *metric* vs. *rhyme* vs. *r&m* for *rhyme+metric,* respectively) and all interactions between the three factors.

In both the base and the main model, intercepts for participants and items were included as random factors. The rationale for this is the different sets of IAs and predictors in both models. Some readers might react to anomalies and layout manipulations differently, resulting in estimate variance, even after general reading measures have had normalized across all IAs. Also, stimulus manipulations might have different effects in different items. Furthermore, slopes for *word length* and *frequency* were added in the base model, and the slope for *MRRL_version* in the main model.

*Variables in the base and complete model.* We included three types of variables in both the base and the complete model: lexical, structural, and oculomotor variables.

*Lexical variables.* We computed five *lexical features*: 1. word category annotated *cat* (labeled *catC*, *catA*, *catN*, *catV*; which identified levels *closed class,* *adverb/adjective, noun*, *verb*), 2. *word length*, i.e., the number of characters for each word (*word_length)* and 3. *log word frequency* (*log.freq*) based on the DeReWo-2014 corpus-based word lists (11).

We computed 4. the *consonant vowel quotient* (*cvq*), as an indicator of pronounceability (78; 85; 114 ; 149). The calculation was based on letters rather than sounds. For German, a high level of consonants is assumed to impede pronunciation, as can be experienced in tongue twisters (e.g. “Schlickkrebskriechgang” / ”Schlickkriechkrebs-schleichgang”). We also added the consonant vowel quotient of the succeeding word (cvq.p1) as an indicator of parafoveal processing of phonological/pronunciation information.

Finally, 5. the *number of syllables* (*syllables*) of a word were computed as an estimate of how long it would take to be spoken. Naturally, number of syllables and the number of characters (*word_length*) of words are highly correlated (.84, see table 1). We therefore computed residualized number syllables (*res.syllables*) in a simple regression over word-types, where syllables were predicted from word length. *Res.syllables* is thus independent of word length and reflects pronunciation more purely. In earlier research, syllable number has been shown to influence skipping, but no effect on reading time measures beyond word length was found in normal reading (40 ). Hence, we would consider any such effect in our results a strong indicator for an eye-voice-span synchronization induced by MRRL-language.

**Table 1.** Correlation matrix of lexical variables

Variables	1	2	3	4
1. word_length	—			
2. syllables	.84	—		
3. log.freq	-.58	-.51	—	
4. cvq	.15	-.26	-.06	—

Also, since the *cvq* turned out to be highly negatively correlated with *res.syllables*, we computed the residual cvq (*res.cvq*) by predicting the *cvq* from both *res.syllables* and *word_length* in a linear regression model over word types.

*Structural variables.* In addition, we computed variables related to particular IA-positions that are known to influence reading, such as the beginning (*BOL*) or end of a line (*EOL*).

Furthermore, we included the variables beginning of verse (*BOV*) and end of verse (*EOV*). Although *EOV*s coincide with *EOL*s in poem layout, they do not necessarily do so in prose layout. The ending of a verse signals an end point of an important (rhythmic) unit and could thus influence subvocalization, e.g. by triggering a pause, independent of a visual line break.

We also included *page* number, the running word number on a single page (*wpos*), and the interaction between the two in order to capture adaptation effects throughout reading a complete item. To account for potential practice or fatigue effects we included the variable *trial* (values 1 to 8), encoding the presentation order of trials throughout the experiment, i.e., the position number of each trial in the experiment.

*Oculomotor variables.* To account for potential preview and spill-over effects we included the gaze durations of the predecessor word (*gaze_pre.word*) as a linear predictor. Because first pass duration measures can vary considerably depending on whether first pass reading is followed by a regressive saccade, we also added the binary predictor *first_pass_regression*.

**Eye tracking parameters.** Before we computed eye-tracking measures from the fixation reports, all single fixations on an IA shorter than 40 milliseconds were treated as overshoots and assigned to the previously fixated IA. Data cleaning, including outlier elimination, was done completely automatically. For each IA, we computed *first fixation durations* (*FFD*), *single fixation durations* (*SFD*; equaling *FFDs*, but excluding all cases with more than one fixation during first pass), *gaze duration* (*GAZE*, the sum of all fixations on the target IA during first pass), *regression path duration* (*RPD*), the sum of all fixation durations during first pass plus - if the first pass is followed by a regressive saccade - all fixation durations on predecessor IAs, until a saccade goes past the target IA (72), right bounded reading time (*RBRT*, the sum of all fixation durations on the target IA until a saccade goes past the IA), *total reading times* (*TRT*, the sum of all fixations on an IA), and *second pass reading time* (*SPRT*, computed as *TRT* minus *GAZE*). All first pass measures (*SFD*, *FFD*, *RD*, and *RBRT*) required the first fixation resulting from a progressive saccade. Also, we analyzed conditionalized times, meaning that zero values were treated as missing values. For data analysis, all time-based parameters were logarithmized.

In addition to these reading time measures, we computed variables coding whether or not a word has been skipped (*SKIP*).

Before model fitting, we calculated overlaps and correlations between the eye tracking parameters (see table 2). Because single fixation durations (*SFD*) are a subset of first fixation durations and first pass reading times, their correlation must equal 1. All other measures – with the exception of *SPRT* (second pass reading times) and both *SFD* and *GAZE* – are significantly correlated with each other (p<.001), albeit to a varying degree.

**Table 2.** Correlations between common eye-movement parameters.

Variables	1	2	3	4	5	6	7
1. SFD	—						
2. FFD	1	—					
3. GAZE	1	.47	—				
4. RPD	.06	.08	.21	—			
5. RBRT	.41	.33	.74	.51	—		
6. TRT	.28	.25	.57	.42	.78	—	
7. SPRT	.01	.01	.03	.28	.49	.87	—

Single and first fixations are a subset of fixations that constitute gaze durations, and therefore their correlation is 1. However, since single fixation durations (*SFD*) and *GAZE* share only 74.7% of the data points, we will report results from both model fits. First fixation durations (*FFD*), on the other hand, will be ignored. Right bound reading times (*RBRTs*) are highly correlated with regression path durations (*RPD*), so they will be ignored, too. We also ignored second pass reading times (*SPRTs*), because they are highly correlated with total reading times (*TRT*, .87). The remaining measures should suffice to tap into early and later processing stages.

Total reading times are a combined measure of first pass and later processing. Therefore, there will be an overlap with *GAZE* and single fixation durations (*SFD*), but any deviations would suggest later stage processes. Total reading times (*TRT*) are thus considered a measure of overall processing difficulty.

Finally, we computed *Load Contributions* (71 ) as a measure of selective re-reading. *Load contributions* (*LC*) measures the time spent re-reading (sum of all fixations on) a previous region in the regression path of a later region. This measure is of particular relevance, because we are interested in whether the eyes re-fixate the pre-rhymes in cases of meter and rhyme anomalies.

Before each stage, and for each response duration variable, extreme values were eliminated. We first identified extreme values by using the function boxplot() with range 3. Hence, outliers were defined as values beyond the most extreme data point which is no more than three times the inter-quartile range from the box.

Then we fit the base model (stage 1), and again - in the same way - identified and eliminated extremes in the residuals. The base model was fit a second time and the resulting residuals were finally merged back into the dataset. From here on, only the critical interest areas were used to fit the main model.

For duration variables, we fit linear mixed effects regression models, using the function *lmer()* from the *lme4 R-*package (version 1.1-21; 9, p. 4). The binary variable skip was analyzed with *logistic* mixed effects regression, using the function *glmer()*.

For all model fits, we used *sum contrast coding*, creating predictors for all but the last level of any categorial variable and assigning 1 to the corresponding level for each comparison as well as -1 to the last level for all comparisons. Remaining levels were coded 0 (*table 3*).

In *sum coding*, the intercept represents the grand mean, and each contrast represents a comparison of a factor level mean to the grand mean. Therefore, all effects are independent of each other. Hence, simple contrasts can be interpreted similar to main effects in ANOVAs – even when the predictor also occurs in interaction terms in the model.

**Table 3.** Sum contrast coding for variable anomaly_type.

	metric	rhyme
Metric	1	0
Rhyme	0	1
r&m	-1	-1

P-values for linear mixed models were estimated with Satterthwaite's approximation of degrees of freedom, using the *lmerTest R-*package (version 3.1-0, (81).

We will first present reading time results, starting with the *main model* and continuing with the *complete model*. *Skipping* results will be presented next, and lastly, we will present *Load Contribution* results.

**Extreme values**. For *single fixation durations*, seven extreme values were filtered from raw data (0.02%). Additional 32 data points (0.08%) were eliminated as outliers from the complete model, and 35 (0.09%) from the base model. No extreme values were excluded after fitting the main model.

For *gaze durations* (first pass reading times), seven extreme values were filtered from raw data (0.02%). No extreme values were excluded from the data set for the base, the complete and the main model.

For *regression path durations*, 32 extreme values were filtered from raw data (0.08%), 10 data points (0.03%) were eliminated as outliers from the complete model, 13 data points (0.03%) from the base model. No extreme values were excluded from the dataset for the main model.

For *total reading times*, 2 extreme values were filtered from raw data (0%). One additional data point (0%) was eliminated as outlier from the complete model, and 2 data points (0%) were as outliers eliminated for the base model. No extreme values were excluded from the dataset for the main model.

## Results

***Main Model**.* We predicted that metrical anomalies induce disruptions, if the metrical structure of the poems was recognized and the anomaly was hence experienced as diverging. We also predicted that the poem layout facilitates the capturing of the rhythmic gestalt, enhancing potential effects of metrical anomalies.

**Figure 5.**
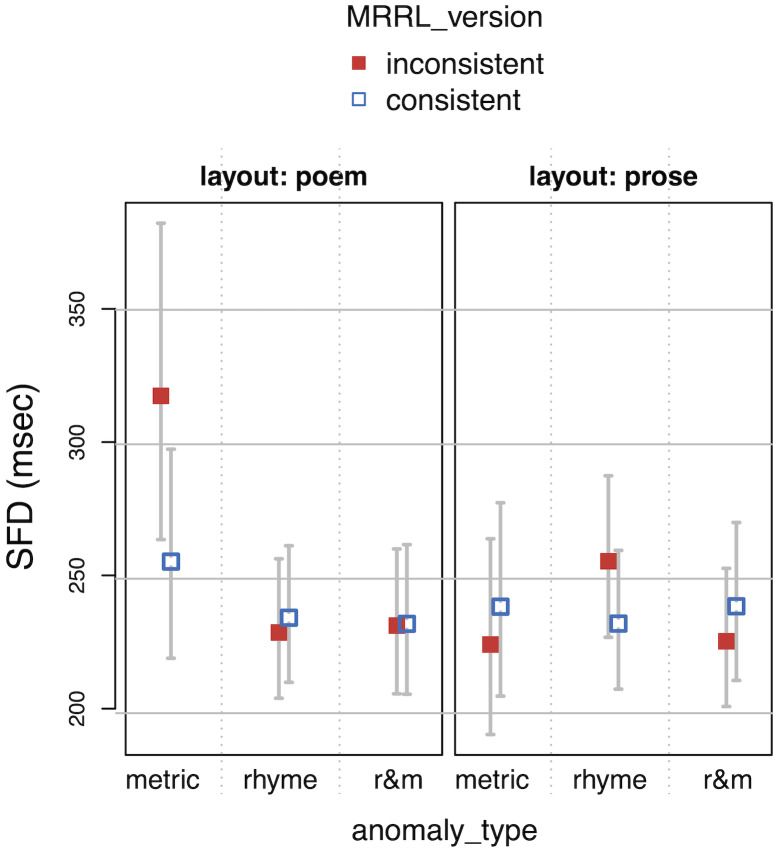
Single fixation durations (SFD) as a function of layout, MRRL_version and anomaly_type.

**Figure 6.**
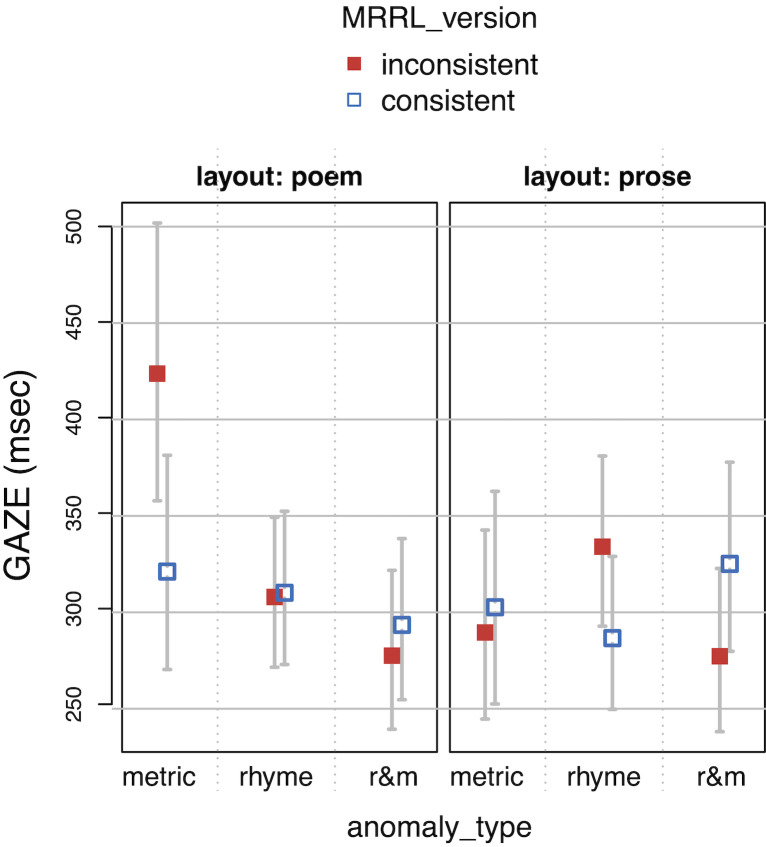
Gaze durations (GAZE) as a function of layout, MRRL_version and anomaly_type.

**Figure 7.**
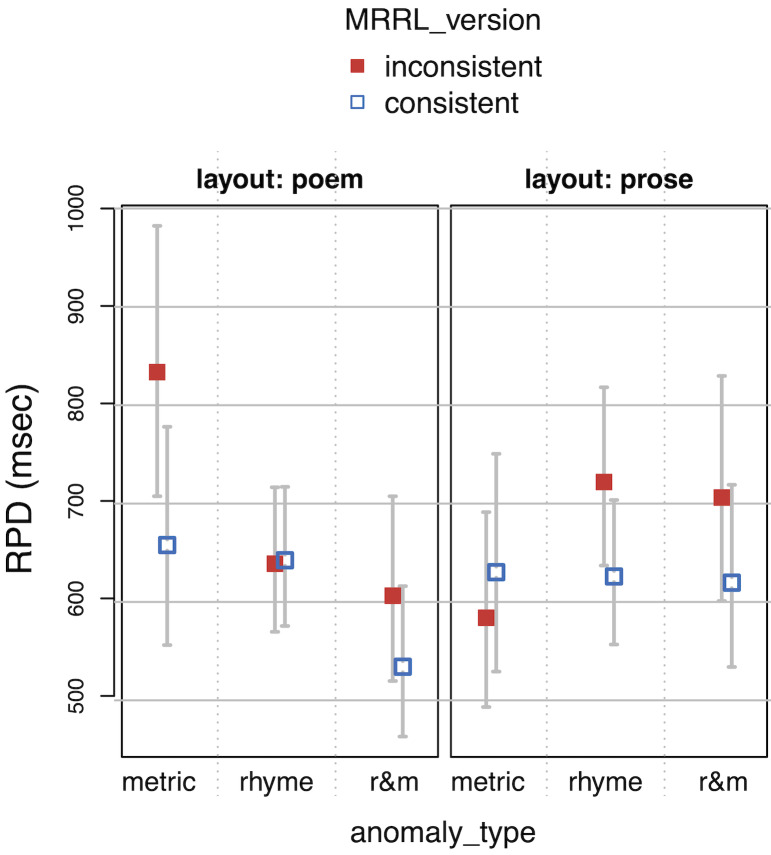
Regression path duration (RPD) as a function of layout, MRRL_version and anomaly_type.

**Figure 8.**
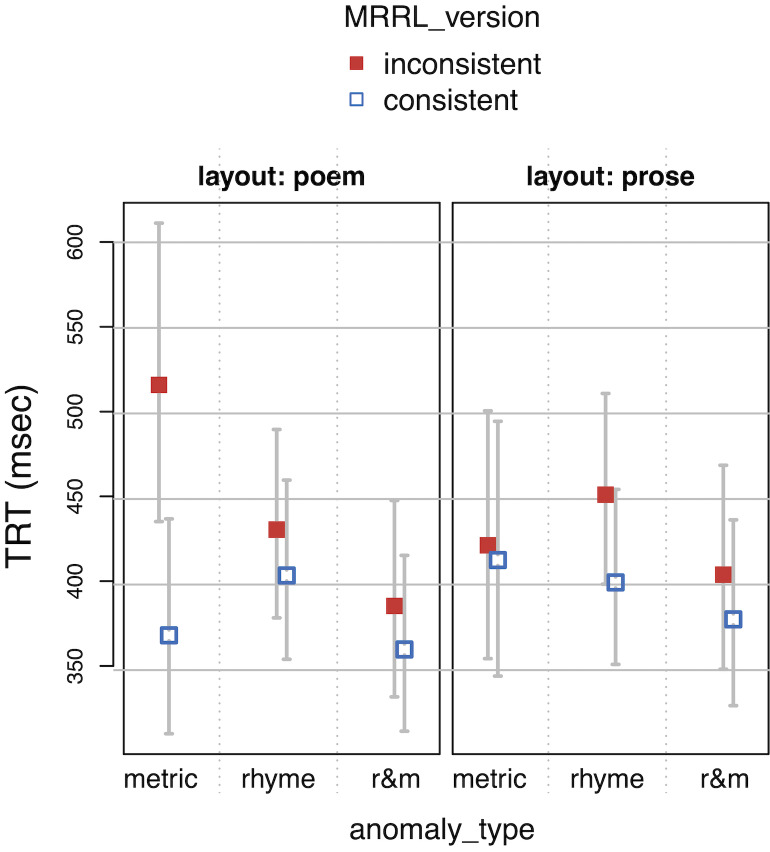
Total reading time (TRT) as a function of layout, MRRL_version and anomaly_type.

The results of the linear mixed effects model fits of the *main model* (table 4) show a fairly robust pattern across eye-tracking measures (see *figure 5, figure 6, figure 7, figure 8*). In poem layout, metric anomalies resulted in increased fixation and reading times compared to the metrically consistent version. This amounts to a significant three-way interaction of factors *layout*, *MRRL_version*, and *anomaly-type* *metric* for measures *GAZE*, *RPD*, and *TRT (SFDs* were only marginally reliable: p=.066 - two way test), on top of the two way interaction of *layout* and *anomaly_type metric* for measures *SFD*, *GAZE* and *RPD*, and a main effect of *MRRL_version* for measures *RPD* and *TRT*.

Post-hoc contrasts between *inconsistent* and *consistent* *MRRL_versions* of *metrical* anomalies in the *poem* layout turned out to be significant for *SFDs* (directional hypothesis), *GAZE,* *RPD*s, and *TRTs* (see table A). Also, in the *poem layout* (table C), post hoc contrasts between inconsistent MRRL_versions of *metric* and *rhyme* anomalies (*SFD*, *GAZE*, *RPD*), as well as *metric* and *r&m* anomalies turned out to be significant (all measures). *Metric anomalies* (*MRRL_version inconsistent*) also elicited longer reading time in *poem* than in *prose layout* (see table B, all measures).

We also expected effects of rhyme violations to be stronger in poem layouts, if the layout was necessary to recognize the poetic form. On the other hand, identifying the pre-rhymes may be more demanding in prose layout, where visual cues to their positions are lacking.

In *prose layout*, *rhyme* anomalies notably triggered longer reading times, resulting in reliable three-way interactions of *layout*, *MRRL_version*, and *anomaly_type rhyme* in *SFDs*, *GAZE*, and *RPDs*, on top of a two-way interaction between *layout* and *anomaly_type rhyme* for *SFDs*, and the aforementioned main effect of *consistency* (*MRRL_version*). Simple post-hoc contrasts between the *inconsistent* and *consistent* version in that condition were significant (directional hypothesis) for gaze durations and *RPDs* (see table A). Combined *metric* and *rhyme* (*r&m*) anomalies (*MRRL_version inconsistent*) in *prose layout* elicited smaller *GAZE* durations but larger *RPDs* than their *consistent* counterparts. This pattern suggests that *r&m* anomalies in prose layout triggered early regressive saccades during first pass reading, resulting in shorter *GAZE* durations and longer *RPDs*.

**Table 4.**
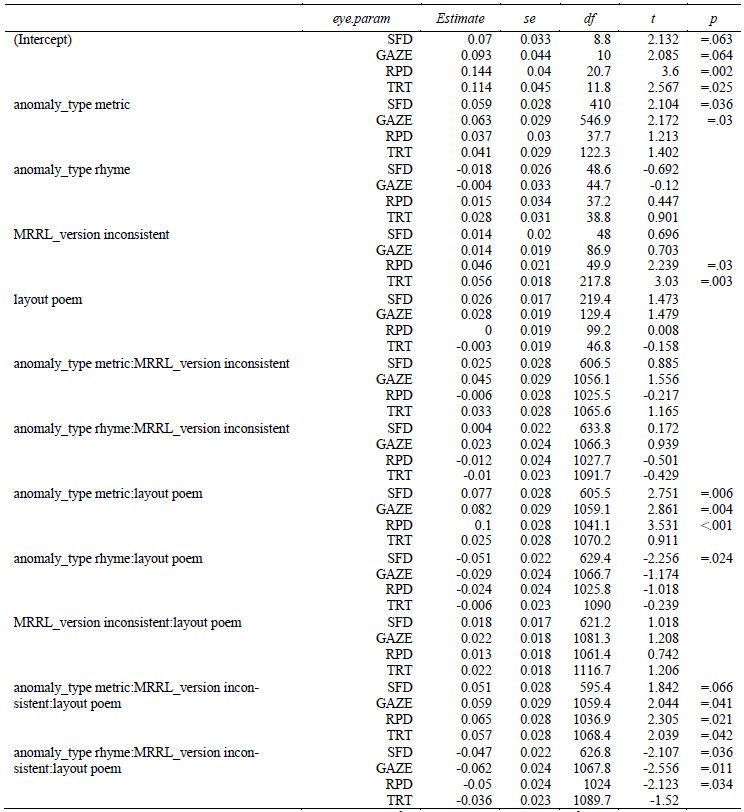
Linear mixed effects regression coefficients of the main model. Time measures are residualized and logarithmized.

Note. For SFD, the number of observation was 699, the conditional R^2^ was 0.118 and the marginal R^2^ was 0.0213. For GAZE, the number of observation was 1143, the conditional R^2^ was 0.129 and the marginal R^2^ was 0.0246. For RPD, the number of observation was 1125, the conditional R2 was 0.176 and the marginal R2 was 0.0265. For TRT, the number of observation was 1188, the conditional R2 was 0.127 and the marginal R2 was 0.0188. Model specification: res.LOG.<duration> ~ anomaly_type * MRRL_version * layout + (1 | vp) + (0 + MRRL_version + layout + anomaly_type | vp) + (1 | item)

Post-hoc contrasts between *layout poem* and *prose* for combined *metric* and *rhyme* (*r&m*) anomalies (see table B) show shorter *RPDs* for layout poem for both versions (*consistent*/*inconsistent*) and overall longer ones for *layout prose*. One possible explanation is that this result is an artefact which can be traced back to the positions of the *r&m* anomalies. First, because of the presentational conditions (see *figure 3* or *Appendix*), in *prose layout,* *r&m* anomalies appeared on page 2. As a result, here, readers were confronted with more text material, which could have elicited longer *RPDs* for both, *MRRL_version inconsistent* and *consistent*. Second, because in poem stimuli presentation readers were not able to jump back from page 3 to page 2, in the *poem layout*, less text material could be re-read. This is applicable for both versions (*consistent*/*inconsistent*) and most likely accounting for the shorter *RPDs* compared to prose layout.

**Table 5.** Main model post-hoc contrasts. Time measures are residualized and logarithmized

*MRRL_version (A)*	*contrast*	*eye.param*	*Estimate*	*SE*	*df*	*t.ratio*	*p*
layout = poem, anomaly_type = metric	inconsistent - consistent	SFD	0.2153	0.1194	362	1.804	=.072
		GAZE	0.27800	0.1108	813	2.509	=.012
		RPD	0.23684	0.1094	746	2.164	=.031
		TRT	0.3350	0.1054	867	3.179	=.002
layout = prose, anomaly_type = rhyme		GAZE	0.15293	0.0831	514	1.840	=.066
		RPD	0.14291	0.0826	433	1.729	=.085
layout = prose, anomaly_type = r&m		GAZE	-0.15909	0.0780	458	-2.039	=.042
		RPD	0.13070	0.0783	387	1.670	=.096
*layout (B)*	*contrast*	*eye.param*	*estimate*	*SE*	*df*	*t.ratio*	*p*
anomaly_type = metric, MRRL_version = inconsistent	poem - prose	SFD	0.34350	0.1218	406	2.821	=.005
		GAZE	0.381739	0.1058	818	3.608	<.001
		RPD	0.3561	0.1045	807	3.408	<.001
		TRT	0.2485	0.1036	811	2.400	=.017
anomaly_type = r&m, MRRL_version = inconsistent		RPD	-0.1525	0.0758	461	-2.011	=.045
anomaly_type = r&m, MRRL_version = consistent		RPD	-0.1484	0.0788	501	-1.884	=.060
*anomaly_type (C)*	*contrast*		*Estimate*	*SE*	*df*	*t.ratio*	*p*
MRRL_version = inconsistent, layout = poem	metric - rhyme	SFD	0.323769	0.1056	248	3.065	=.007
		GAZE	0.3197	0.0968	288	3.303	=.003
		RPD	0.26612	0.0964	255	2.762	=.017
	metric - r&m	SFD	0.312746	0.1040	244	3.006	=.008
		GAZE	0.4242	0.0944	343	4.496	<.001
		RPD	0.31847	0.0943	303	3.379	=.002
		TRT	0.28156	0.0906	373	3.106	=.006

The whiskers for figure 5-8 indicate 95% confidence intervals. Raw reading times were back-transformed from residual logarithmized model estimates by first adding the base model intercept and then applying the exp-function.

### Discussion

For the *main model*, all four eye-tracking measures (see *figure 5, figure 6, figure 7, figure 8*) revealed a high sensitivity to the *metric anomaly* in poem layouts, resulting in increased fixation and reading times. This finding suggests that the poem layout is mandatory to detect the metric anomaly.

Thus, when participants read poems, they pick up the overall prominent (linguistic) metrical grid, and their expectations get disrupted when the metric scheme is broken. Increased reading times on the word that introduces the anomaly in poem layouts thus indicate that readers apparently look for a solution on that very word. This is a clear early indicator for rhythmic subvocalization.

Interestingly, the rhyme anomaly effect for *GAZE* and *RPD*s in prose layout suggests that readers were expecting rhyme words to some extent, presumably elicited by the MRRL-structure. Here, rhyme anomalies have resulted in hesitations that signal disrupted expectations. In poem layout, however, rhyme anomalies have not disrupted reading, presumably because the strict poetic form facilitated the adoption of a different, yet common, rhyme scheme ABAC.

*R&m anomalies* in prose layout lead to shorter *GAZE* durations but longer *RPD*s, indicating that specifically the combined anomaly triggered early regressive saccades during first past readings. The seemingly contradicting results in this condition for *GAZE* and *RPD*s highlight the importance of interpreting eye tracking measures in relation to each other and not independently from one another.

### Complete Model

*Generic variables.* As expected, more frequent words (*log.freq*) elicited significantly shorter reading times in all four variables (*SFD, GAZE*, *RPD,* and *TRT,* see *table 6*). *Word_length* also showed a significant increase of reading times for longer words in all variables except *SFD*s.

Among the pronunciation-related variables, we found a significant effect of residual number of syllables (*res.syllables*) in all four measures. The effect indicates a strong impact of subvocalization on reading. Even *SFD*s, which showed no reliable effect of word_length, were significantly increased for *res.syllables,* suggesting a closer link of *SFD*s to pronunciation rather than to visual word processing in our study.

Number of syllables (*res.syllables*) did also interact with MRRL-version in early measures (*SFD* and *GAZE*) indicating that words with more syllables were fixated even longer when anomalies were present in the poem.

**Figure 9.**
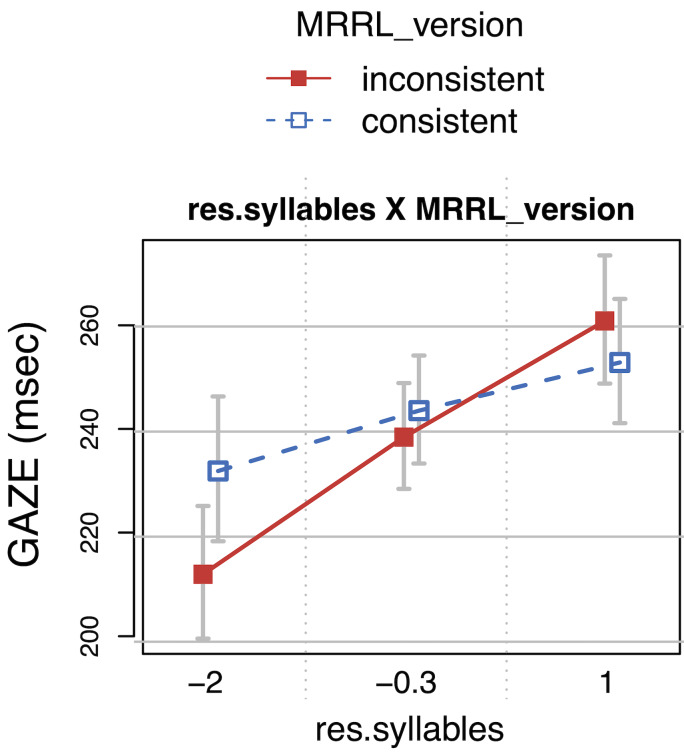
Gaze durations (GAZE) as a function of (residual) number of syllables and MRRL_version. The whiskers indicate 95% confidence intervals. Raw reading times were back-transformed from the logarithmized estimates.

The residual *consonant vowel quotient* (*res.cvq*), included as another indicator of subvocalization, did not turn out significant nor did any interaction with *res.cvq*.

Reliable effects of *first_pass_regression* indicate shorter *SFD*s and *GAZE* durations, whenever first pass reading ended in a regressive saccade, consequently increasing *RPD*s and *TRT*s. Line endings (*EOL*+) were read reliably faster in all four measures, whereas line beginnings (*BOL*+) were read slower than words in other line positions. Reliable effects of *trial* and *wpos* in *TRT*s indicate a speed-up for later experimental trials and throughout a single page, respectively, suggesting a practice effect or adaptation. The acceleration of *TRT*s on a single page (*wpos*) was even stronger on later pages, as indicated by a reliable interaction of *wpos* and *page.*

One might argue that the speed up towards the end of a trial contradicts the principle of isochronicity. In our view, this is not the case. One can read/recite ’empirically isochronal‘ (112, 111 ) along with a metrical grid and speed up or slow down reading tempo, as long as the inferred ’beat‘ is evenly distributed in a certain time window (similar to speeding up/slowing down in musical piece), such as a verse or a stanza.

Word *category* also showed reliable effects in all measures. Effects of these generic variables were to be expected and confirm the accuracy and soundness of our measurements.

Higher gaze durations of the previous word (*gaze_pre.word*) elicited a positive effect on single fixation durations, suggesting a short processing spill-over as well as a negative effect on the *GAZE*, *RPD*s and *TRT*s suggesting a preview effect.

**Figure 10.**
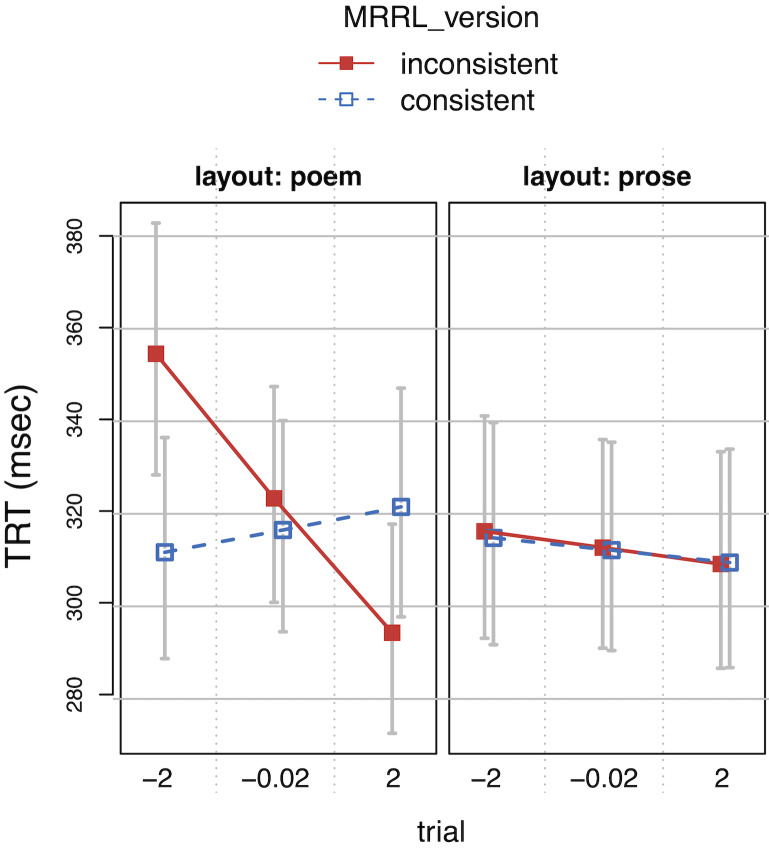
Total Reading Times (TRT) as a function of *layout*, *MRRL_version* and *trial* (centered). The whiskers indicate 95% confidence intervals. Raw reading times were back-transformed from the logarithmized estimates.

While the presence of anomalies (*MRRL_version inconsistent*) did not alter reading over all, measures representing late processing, *RPD*s and *TRT*s, showed significantly slower reading in *poem layout*, and a slight acceleration in *TRT*s towards the end of the experiment (interaction *layout* by *trial*), in particular for inconsistent poem versions (interaction *MRRL_version* by *layout* by *trial*). TRTs apparently reflect the adaptation to the design manipulations very well (*figure 10*).

**Figure 11.**
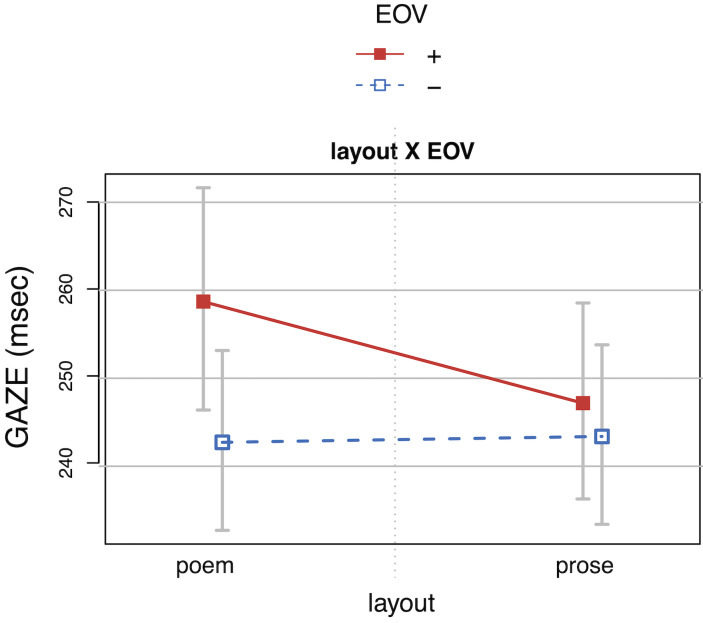
Gaze duration (GAZE) as a function of layout, and end-of-verse (EOV). The whiskers indicate 95% confidence intervals. Raw reading times were back-transformed from the logarithmized estimates.

Verse endings (*EOV*+) showed a significant increase in all four reading time measures, indicating that verse endings were processed as *MRRL* grouping cues independent of line breaks. However, the effect was carried by the poem layout, where verse and line endings coincide (interaction *layout* by *EOV;* see *figure 11* for gaze durations; SFDs and RPDs show a similar pattern). This finding is partly in line with Fechino et al. (36), who report an interaction *verse last word* by *visual presentation* for *first fixation and gaze duration*). Moreover, the presence of anomalies (*MRRL_version inconsistent*) increased *SFD*s and *TRT*s at verse endings (interaction *MRRL_version* by *EOV*).

Verse beginnings (*BOV*+) showed faster *SFD*s and *TRT*s, but slower *RPD*s independent of line beginnings. In *prose* layouts, *BOV*s showed increased *SFD*s, *GAZE* durations, and *TRT*s, compared to *BOV*s in *poem* layouts, where they coincide with *BOL*s. If reading times mirror pronunciation times, the effect may either indicate additional pausing for in-line *BOV*s or decreased pausing for verse beginnings at the beginning of lines. The presence of anomalies (*MRRL_version consistent*) elicited faster *TRT*s at verse beginnings. Taken together with the reversed effect at verse endings (*EOV*+), this pattern of results suggest that anomalies draw the attention to verse endings, at the expense of verse beginnings in later processing stages, as measured by *TRT*s. The *consonant vowel quotient* of the next word (*cvq.p1*) was added to establish potential parafoveal effects of pronounceability. At line endings (*EOL+*), where parafoveal preview is impossible, only GAZE durations showed significantly decreased values for higher *cvq*s on the next word (*cvq.p1*) indicating their sensitivity to potential preview effects. However, *GAZE* durations were generally smaller for higher *cvq.p1*s. *SFD*s increased with higher a *cvq.p1*, but less so in poem layouts.

### Discussion

The complete model revealed that reading was slowed down in poem layout, but only in late measures (*RPD*s and *TRT*s). Increased RPDs are due to more first pass regressive saccades from the word. TRTs represent refixations to the word. Both indicate that eye progression (moving forward) decelerates, suggesting more cautious reading, and potentially maintaining a narrow eye to voice span. Furthermore, rhythmic subvocalization supposedly puts an emphasis on constantly updating local stress patterns, requiring the eyes not to jump too far ahead of the inner voice and, at the same time, force the reader into revisiting of the immediate preceding word-material. This would ultimately result in an increase of local regressive saccades, and, hence in elevated *RPD*s and *TRT*s.

*TRT*s showed interesting results in overall reading. Note that critical IAs were not included in the overall analysis (*complete model*). Reading speed increased in later trials, however mostly when anomalies were present in poem layouts. Readers were disrupted by anomalies more strongly in the poem layout at the beginning of the experiment, but got used to them in later trials, while readers basically kept the same pace throughout the entire experiment otherwise (*figure 10*).

A syllable is a single unit of speech. The complete model established the number of syllables of a word as a strong indicator of subvocalization in silent reading of poetry. Because the word length has been factored out beforehand, the residual number of syllables (*res.syllables*) represents pure pronunciation length. Hence, when word reading time increases with its number of syllables, it directly reflects the pronunciation duration of a word and is thus closely linked to subvocalization, suggesting a very narrow eye-to-(inner)-voice span.

All reading time measures were sensitive to syllables. Only for single fixation durations did word length *not* elicit a significant effect. While SFDs are usually sensitive to lexical and visual characteristics of words, these were obviously dominated by properties of pronunciation in our study. This might be due to specific task demands of our study, namely just reading MRRL-poetry without any requirement for comprehension, and seems to indicate that participants resorted to a more shallow processing mode, while they were focusing on the ‘sound of the language’ and its musical quality.

Somewhat surprisingly, the residual consonant vowel quotient *res.cvq* did not affect reading at all. The *cvq* supposedly represents the pronounceability of words, under the assumption that a higher consonant density leads to impoverished speakability. However, word-*cvq* might be just one, and possibly a minor factor of many contributing to (un-)speakability, such as slight divergencies of otherwise similar syllables in the immediate context (*Brautkleid bleibt Brautkleid*).

More importantly for this study: the *cvq* implicitly represents *syllable length*, as syllables are constructed around vowels as their nucleus, which is surrounded by a varying number of consonants. The *cvq* is thus mainly determined by the number of consonants per syllable, and therefor represents, when calculated per word, its average syllable length. Consequently, we would expect collinearities of the three variables *word_length*, *number of syllables*, and *cvq*, rendering the latter virtually redundant.

This conclusion is corroborated by our finding that in our materials, the residual number of syllables *res.syllables*, where word length is factored out, and the *cvq* were highly negatively correlated (r=-.71). Accordingly, the non-effect of *res.cvq* is no surprise. Of course, this does not mean that the *cvq* might never represent pronounceability in other materials. Whether or not it does depends on other pronunciation-related features in the particular study material captured by the *cvq*, such as sequences of consonants that are actually difficult to pronounce. In our materials however, the *cvq* did not contribute anything on top of *residual number of syllables*.

It hence remains unclear, whether the effects of the *cvq* of the successor word (*cvq.p1*) indicate preview effects of pronounceability, since we did not control for *the residual number of syllables* of the successor word. Nevertheless, the preview effect is an indicator of some

lexical, and probably pronunciation related, pre-processing of the successor word. As such, it strengthens the notion of a narrow eye to (inner) voice span.

**Table 6.**
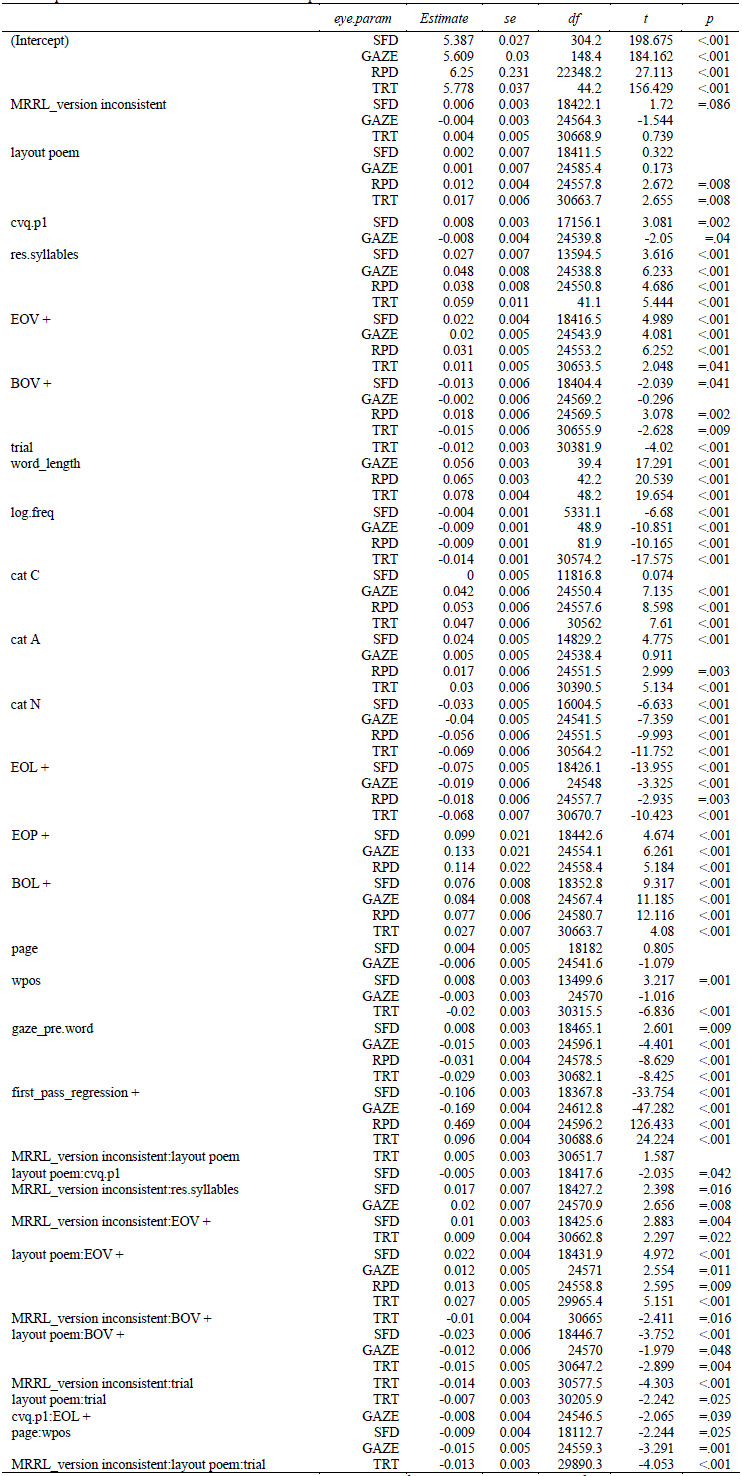
*Complete model*. Estimates are based on logarithmized measures. Reading time measures (dependent variables) are only listed for predictors that remained in the model after stepwise elimination.

Note. For SFD, the number of observation was 18505, the conditional R^2^ was 0.159 and the marginal R^2^ was 0.092. For GAZE, the number of observation was 24659, the conditional R^2^ was 0.242 and the marginal R^2^ was 0.172. For RPD, the number of observation was 24638, the conditional R^2^ was 0.51 and the marginal R^2^ was 0.448. For TRT, the number of observation was 30765, the conditional R^2^ was 0.279 and the marginal R^2^ was 0.149. EOV + stands for *end-of-verse* = true, BOV stands for *beginning of verse*, EOL stands for *end of line*, BOL stands for *beginning of line, wpos* represents the *serial order position of a word on a page.* For further information, see section *predictors* in *data analysis.* Model specification: log.<duration> ~ MRRL_version * layout * res.cvq + MRRL_version * layout * cvq.p1 + MRRL_version * layout * res.syllables + MRRL_version * layout * EOV + MRRL_version * layout * BOV + MRRL_version * layout * trial + word_length + log.freq + res.syllables + res.cvq + cvq.p1 + cat + EOL + cvq.p1:EOL + EOP + BOL + page * wpos + gaze_pre.word + trial + first_pass_regression + (1 | vp) + (0 + res.syllables + word_length + log.freq | vp) + (1 | item)

**Skipping probability.** Across all conditions, words were skipped with an average rate of .24 (*sd* = 0.16). The most frequently skipped words were short function words and pronouns at the beginning of a new line (e.g., *Er*, *Am*, *So*, *Da*), with a skipping probability up to .88. Twenty words were never skipped at all (e.g., *aufgezehrt*, *Chorusgleis*, *denkt*, *Feuertopf*).

For skipping probability, we did not fit the main model for the critical IAs, because almost no skipping was found here. However, we fitted the complete model. Due to the binary nature of the response variable, we fitted a logistic regression using a similar predictor structure (excluding *cvq.p1* and including the interactions *word.length:BOL*, *log.freq:BOL*, *cat:BOL*, *layout:BOL, page:layout*, *wpos:layout*) of the complete model in earlier fits.

**Table 7.** Skipping probability.

	*Estimate*	*se*	*z*	*p*
(Intercept)	-0.599	0.198	-3.025	=.002
MRRL_version inconsistent	-0.004	0.023	-0.162	
layout poem	-0.066	0.030	-2.173	=.03
res.cvq	-0.075	0.018	-4.260	<.001
res.syllables	0.079	0.040	1.971	=.049
EOV +	0.091	0.026	3.542	<.001
BOV +	0.044	0.162	0.271	
trial	0.059	0.013	4.560	<.001
word_length	-0.386	0.016	-24.477	<.001
log.freq	0.005	0.006	0.870	
cat C	0.243	0.041	5.939	<.001
cat A	-0.041	0.038	-1.068	
cat N	-0.135	0.051	-2.658	=.008
EOL +	-0.350	0.031	-11.297	<.001
EOP +	-0.043	0.156	-0.273	
BOL +	0.809	0.163	4.968	<.001
page	0.144	0.024	6.117	<.001
wpos	-0.020	0.013	-1.500	
gaze_pre.word	-0.092	0.014	-6.446	<.001
MRRL_version inconsistent:layout poem	-0.027	0.023	-1.200	
MRRL_version inconsistent:res.cvq	-0.011	0.017	-0.634	
layout poem:res.cvq	-0.015	0.017	-0.867	
MRRL_version inconsistent:res.syllables	0.006	0.038	0.160	
layout poem:res.syllables	0.068	0.038	1.775	=.076
MRRL_version inconsistent:EOV +	-0.017	0.019	-0.868	
layout poem:EOV +	0.131	0.025	5.240	<.001
MRRL_version inconsistent:BOV +	-0.015	0.016	-0.933	
layout poem:BOV +	-0.221	0.162	-1.366	
MRRL_version inconsistent:trial	0.005	0.014	0.385	
layout poem:trial	0.022	0.014	1.565	
page:wpos	-0.013	0.023	-0.590	
word_length:BOL +	-0.095	0.016	-6.084	<.001
log.freq:BOL +	-0.015	0.006	-2.559	=.01
cat C:BOL +	0.120	0.040	2.976	=.003
cat A:BOL +	0.099	0.038	2.614	=.009
cat N:BOL +	-0.221	0.050	-4.406	<.001
layout poem:BOL +	0.086	0.162	0.531	
layout poem:page	0.060	0.024	2.529	=.011
layout poem:wpos	0.049	0.013	3.661	<.001
MRRL_version inconsistent:layout poem:res.cvq	0.002	0.017	0.096	
MRRL_version inconsistent:layout poem:res.syllables	-0.077	0.038	-2.027	=.043
MRRL_version inconsistent:layout poem:EOV +	-0.036	0.019	-1.892	=.058
MRRL_version inconsistent:layout poem:BOV +	0.018	0.016	1.126	
MRRL_version inconsistent:layout poem:trial	0.041	0.014	2.937	=.003

Note. For skipping probability, the number of observation was 37221, the conditional R^2^ was 0.283 and the marginal R^2^ was 0.0885. EOV + stands for *end-of-verse* = true, BOV stands for *beginning of verse*, EOL stands for *end of line*, BOL stands for *beginning of line, wpos* represents the *serial order position of a word on a page.* For further information, see section *predictors* in *data analysis.* Model specification: skip ~ MRRL_version * layout * res.cvq + MRRL_version * layout * res.syllables + MRRL_version * layout * EOV + MRRL_version * layout * BOV + MRRL_version * layout * trial + word_length + log.freq + res.syllables + res.cvq + cat + EOL + EOP + BOL + page * wpos + gaze_pre.word + trial + word_length:BOL + log.freq:BOL + cat:BOL + layout:BOL + page:layout + wpos:layout + (1 | vp) + (1 | item)

*Generic variables*. The logistic mixed effects regression fit (*table 7*) shows that many variables representing lexical or structural features of words behaved as expected. Skipping probability drastically decreased with word length (*word_length*), while word frequency only affected skipping after the line initial word (*log.freq* by BOL interaction). Skipping was thus generally strongly influenced by shape related visual features, whereas word recognition and lexical access played a role only in parafoveal preview. The reliable effect for *page* shows that skipping increased on later pages of a trial. Skipping was also increased in *poem* layouts (main effect *layout*), particularly towards the end of single pages as well as on later pages, towards the end of poems (*layout poem:wpos*, *layout poem:page*). It also increased with later trials (*trial*) and even stronger when anomalies were present in layout poem (*layout poem:MRRL_version inconsistent:trial*).

**Figure 12.**
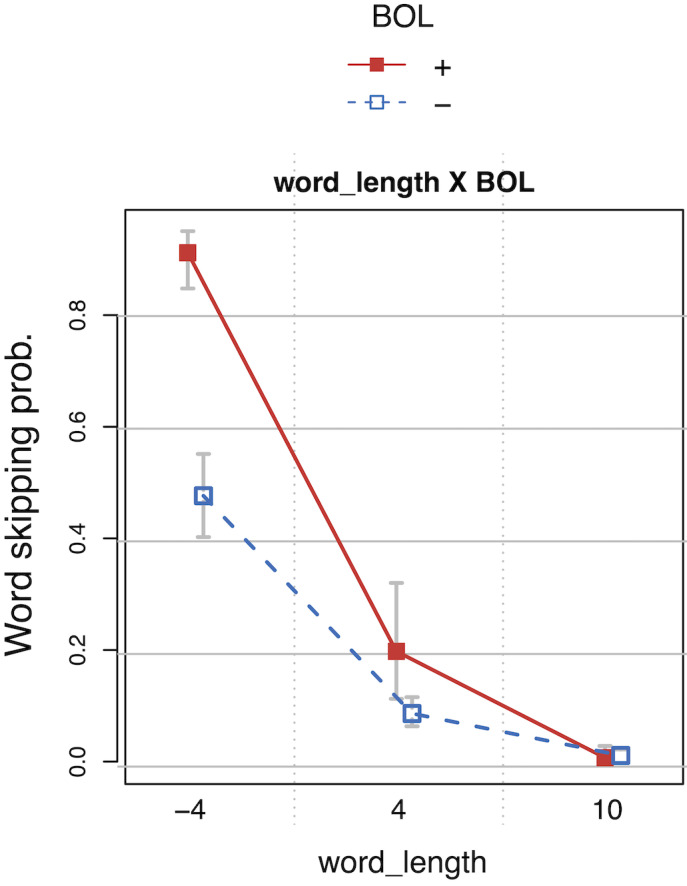
Word skipping probability as a function of word length (*word_length*) and beginning of line (BOL). The whiskers indicate 95% confidence intervals. Word length was centered hence the values range from -4 to 10 with a mean of 4.

Words were skipped significantly more often at beginnings of lines (main effect *BOL+*). The amount of skipping of line initial words was modulated by a variety of lexical variables though: first and foremost, line initial words were skipped much more often when they were very short (*figure 12*).

**Figure 13.**
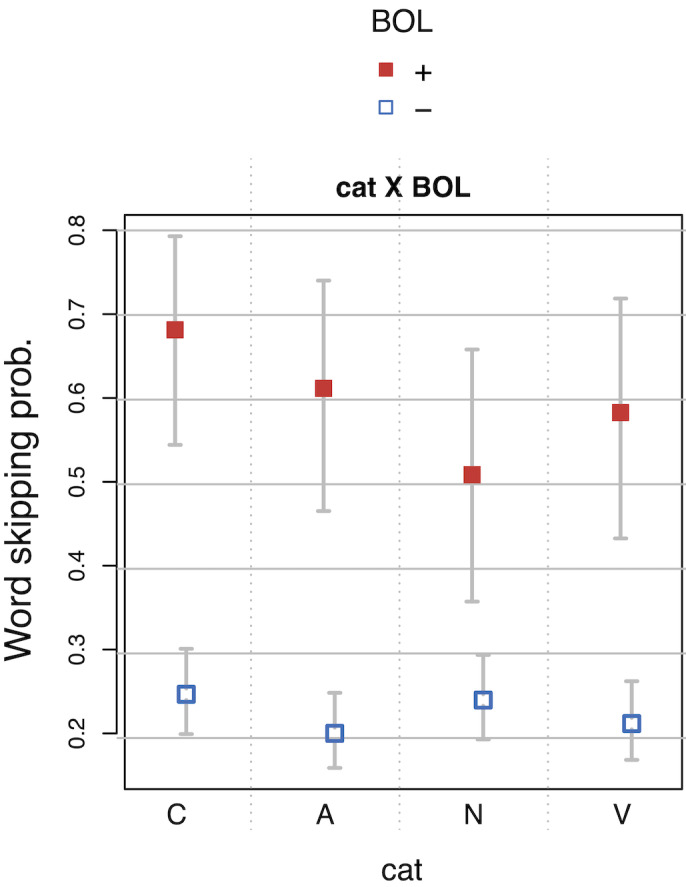
Word skipping probability as a function of word category (*cat*) and beginning of line (*BOL*). The whiskers indicate 95% confidence intervals.

Word category affected skipping as a main effect, but also had a strong impact on skipping the first word of a line (*cat:BOL*). As illustrated in figure 13, nouns were the least likely category to be skipped here, followed by verbs and adjectives. Closed-class words (*cat C*) were skipped most often. Note that this category effect cannot be attributed to the fact that closed class words are short and open class words (*N*, *V*, and adjectives) are longer on average, because *word_length* was taken into account independently, as were other lexical variables.

Interestingly, layout poem did not influence skipping at line beginnings (*layout:BOL*) in addition to the aforementioned variables.

Among the variables related to pronunciation, a higher *consonant vowel quotient* (*res.cvq*) significantly diminished the skipping probability for a word. In layout prose, the residual number of syllables significantly diminished skipping for consistent versions (*MRRL_version:layout:res.syllables*).

While skipping was less likely at the end of lines (*EOL+*), it increased significantly at the end of verses (*EOV*), even more so in the poem layout (*layout poem:EOV*), and marginally so when anomalies were included (*MRRL_version inconsistent:layout poem:EOV*). Verse endings notably coincide with line endings in poem layout, but – with few exceptions – did not so in prose layout. As verse endings are the place where anomalies were realized, it stands to reason that *EOV*s attract more attention in the *inconsistent MRRL_version*.

Skipping probability decreased with larger gaze durations on the previous word (*gaze_pre.word*) suggesting that if the previous word was hard to process, parafoveal preview of the following word might have been limited so that skipping became less likely.

### Discussion

Word-skipping mainly showed expected results with respect to lexical and formal features indicating that skipping in MRRL often mirrors the results of reading times, but also diverges in some respects.

Words at verse ends were skipped more often, and more so in poem layouts.

The presence of anomalies (*MRRL_version inconsistent*) in *poem layout* items, however, sligthly reduced (p=.058) skipping at the end of verses (*EOV+*). Since anomalies always occurred at the end of both verses and lines in *poem layout*, their presence had apparently induced a more cautious reading style at this particular position.

Earlier, we argued that in our study, the *cvq* is highly confounded with average syllable length, which explains the lack of a *cvq* effect on top of a highly reliable effect of residual syllable number in reading times. Nevertheless, we included *res.cvq* in the skipping model because *skipping* might still be more sensitive to pronounceability. In fact, the *res.cvq* significantly decreased skipping, while the residual number of syllables was inconclusive. The differential effects of *res.syllables* and *rec.cvq* make sense though. As syllables are the units of speech, their number should directly affect pronunciation *time*. The probability of a skipping event, however, strongly depends on the word’s length and frequency, i.e., measures of its recognizability and lexical accessibility. Syllable-based pronunciation *duration* might thus add no valuable skipping criterion, whereas pronounceability might very well do. The data support this assumption: skipping was significantly reduced with elevated consonant densities, i.e., higher *res.cvq*s, whereas the number of syllables had virtually no effect on over-all skipping.

The three-way interaction of layout, MRRL-version and syllables, however, mirrors the fact that skipping probability shrinks with the number of syllables only in prose layout when no anomalies were present. This finding suggests that reading in prose layout might have been more cautious and more closely aligned with the inner voice, as long a reading was not disrupted by anomalies.

At line beginnings, *word-length* and word *category* strongly affected skipping, whereas *layout* did not. This finding does not appear to support (15) finding that poem layouts induce more cautious reading. However, he reports the strongest effect for the first function word in text medial position in poem vs. prose layouts. Our BOL effect is mainly driven by word-length and category, with nouns being the least likely words to be skipped. This might appear surprising at first glance, as the category of the line initial word cannot be processed in parafoveal preview from the final words of the previous line. However, word category can often be predicted from the preceding syntactic context. Note that syntactic boundaries are also syntactic prediction cues. Sentences typically start with a determiner or (short) function words such as *and, it,* etc*.,* in particular in our stimuli. Additionally, predictable beginnings in poems are often used as a rhetoric figure of *repetitio*. Hence, readers may be able to statistically predict in a poem, which word may start the beginning of a next line. Line initial word skipping thus appears to depend upon both, bottom-up perceptual features as captured by word length and top-down prediction-based information, such as word category.

### Load contributions of pre-rhymes

The LC measure calculates selective re-reading and allows an investigation of the time spent re-reading (sum of all fixation durations on) a previous region in the regression path of a later region. It helps to indicate whether the

eyes re-fixate the pre-rhymes when a regressive saccade is triggered by a rhyme anomaly. We analyzed an *IA subset* containing all rhyme words, i.e., the last word of the third (A) and fourth verse (B) in each stanza, and computed the time spent on the corresponding pre-rhyme word, i.e., the last word of the first or second verse respectively, when a regressive saccade was launched from the rhyme word after first pass reading.

**Figure 14.**
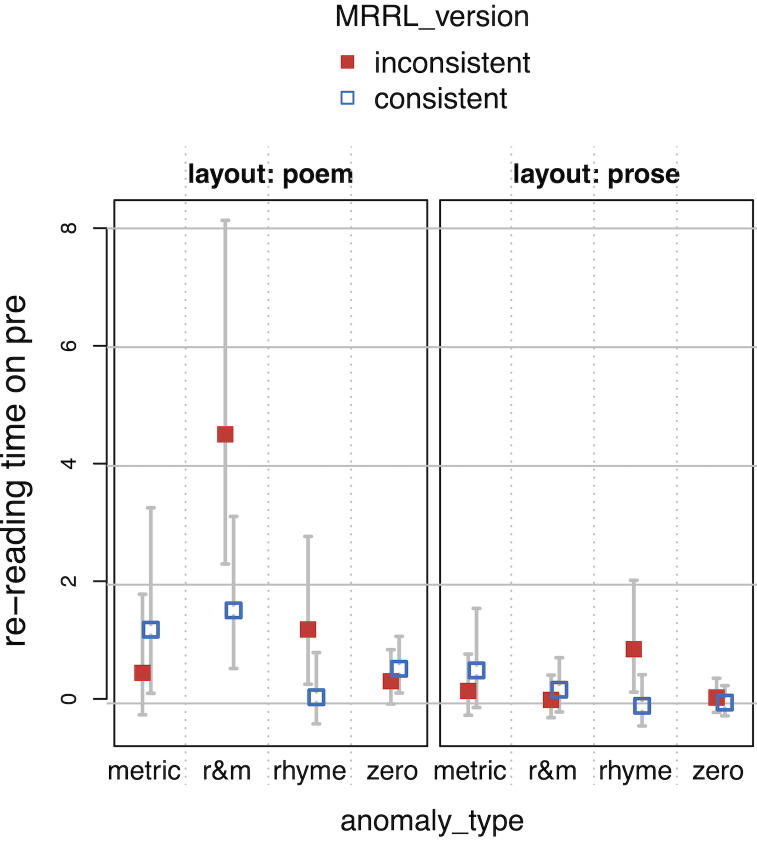
Re-reading time (msec) on prime as a function of *layout*, *version* and *anomaly type*.

The variable *anomaly_type* has been coded only for critical interest areas, which introduced anomalies in the inconsistent MRRL_version. All other rhyme words at non-critical positions were coded *zero*, indicating that the wording was identical for consistent and inconsistent MRRL-versions (*see figure 14*). This allowed us to analyze the effect of rhyme and metric anomalies on the load contributions of pre-rhymes in all stanzas. This *zero* condition, which did not contain any anomalies, served as a baseline condition.

The model fit (table 8) shows that the time spent on a pre-rhyme within a regression path of the corresponding target IA increased significantly when both *metric* and *rhyme* were manipulated in the poem layout (*see figure 14*). The effect amounts to a robust significant three-way interaction of factors *layout*, *MRRL_version* and *anomaly_type r&m* (*anomaly_type r&m:layout poem:MRRL_version inconsistent*), on top of the two-way interactions of *layout poem* and *anomaly_type rhyme and meter* (*anomaly_type r&m* x *layout),* and *layout poem* and *anomaly_type rhyme* (*anomaly_type rhyme:layout poem*), as well as on top of the two-way interaction of *anomaly_type rhyme:MRRL_version inconsistent*, and two significant main effects, one for *r&m* anomalies (*anomaly_type r&m*), and one for *layout poem* (*layout poem*).

For anomaly type *r&m* in *poem* layouts, we found a significant post-hoc contrast between the inconsistent and consistent MRRL_version (*est* = *0.6067, se = 0.220, df = 463, t-ratio = 2.757, p=.006*). For anomaly type rhyme, post-hoc contrast was significant between the inconsistent and consistent MRRL_version in *poem layout* (*est* = *0.5771, se = 0.315, df = 632, t-ratio = 1.832, p=.067*), as well as marginally so in *prose layout* (*est* = *0.5937, se = 0.311, df = 644, t-ratio = 1.911, p=.057*).

**Table 8.** Re-reading time on pre-rhyme within regression path of critical IA.

	*Estimate*	*se*	*df*	*t*	*p*
(Intercept)	0.712	0.189	505.8	3.775	<.001
anomaly_type metric	-0.086	0.124	62.2	-0.696	
anomaly_type r&m	0.429	0.117	105.5	3.651	<.001
anomaly_type rhyme	-0.109	0.114	52.3	-0.964	
layout poem	0.264	0.087	58.8	3.046	=.003
MRRL_version inconsistent	0.064	0.057	59.8	1.126	
wpos	0.264	0.142	1149.8	1.859	=.063
page	-0.204	0.105	1158.6	-1.938	=.053
trial	0.001	0.042	910.2	0.015	
anomaly_type metric:layout poem	0.010	0.115	1106.2	0.083	
anomaly_type r&m:layout poem	0.301	0.075	1171.4	4.031	<.001
anomaly_type rhyme:layout poem	-0.235	0.090	1074.8	-2.615	=.009
anomaly_type metric:MRRL_version inconsistent	-0.177	0.096	1032.8	-1.840	=.066
anomaly_type r&m:MRRL_version inconsistent	0.026	0.069	1136.4	0.382	
anomaly_type rhyme:MRRL_version inconsistent	0.229	0.088	1065.5	2.610	=.009
layout poem:MRRL_version inconsistent	0.029	0.046	1160.8	0.624	
wpos:page	-0.181	0.087	1156.8	-2.075	=.038
anomaly_type metric:layout poem:MRRL_version inconsistent	-0.071	0.097	1007.1	-0.737	
anomaly_type r&m:layout poem:MRRL_version inconsistent	0.184	0.069	1156.9	2.658	=.008
anomaly_type rhyme:layout poem:MRRL_version inconsistent	-0.033	0.087	1137.5	-0.380	

Note. The number of observation was 1243, the conditional R^2^ was 0.288 and marginal R^2^ was 0.076. Model specification: lmer Table: log(lc_pre + 1) ~ anomaly_type * layout * MRRL_version + wpos * page + trial + (anomaly_type + layout + MRRL_version | vp) + (1 | item)

**Figure 15.**
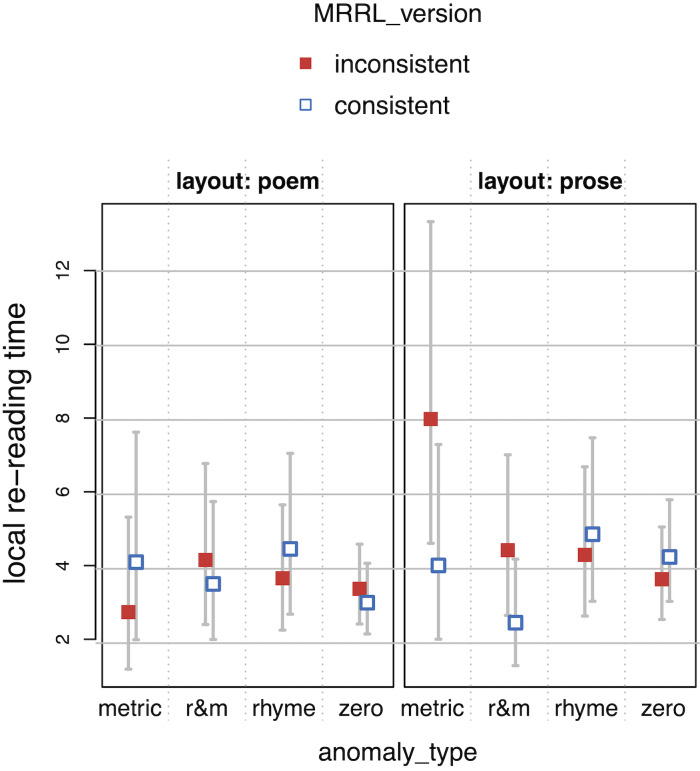
Re-reading time (msec) on *local context* (one to six words before critical IA) as a function of *layout*, *version* and *anomaly type*

We also calculated *load contribution* for the material directly preceding the anomaly. We had predicted that metric anomalies elicit more re-reading the verse that it occurs in, as this is where its rhythmic gestalt is established. This hypothesis seems to be partly supported. The model fit (see table 9, *figure 15*) shows that the time spent on the *local context* (one to six words before critical IA), after a first pass regression was launched from the critical IA, yielded two-way interactions *anomaly_type r&m* x *layout*, and *anomaly_type r&m x MRRL_version,* as well as a three-way interaction *anomaly_type metric* x *layout* x *MRRL_version*.

For anomaly type *metric* in *prose* layouts, we found a significant post-hoc contrast between the inconsistent and consistent MRRL_version (*est* = *0.573, se = 0.241, df = 973, t-ratio = 2.375, p=.0178*). For anomaly type *r&m*, post-hoc contrasts were significant between the inconsistent and consistent MRRL_version, too (*est* = *0.437, se = 0.177, df = 458, t-ratio = 2.472, p=.014*). However, metric anomalies did not induce local re-reading in *poem* layouts as hypothesized.

**Table 9.** Re-reading time (msec) on *local context* (one to six words before critical IA)

	*Estimate*	*se*	*df*	*t*	*p*
(Intercept)	1.352	0.168	178.9	8.049	<.001
anomaly_type metric	0.090	0.106	39.6	0.850	
anomaly_type r&m	-0.076	0.103	80.2	-0.739	
anomaly_type rhyme	0.068	0.085	47.8	0.803	
layout poem	-0.071	0.042	472.1	-1.700	=.09
MRRL_version inconsistent	0.035	0.048	43.6	0.718	
wpos	-0.448	0.107	5562.4	-4.183	<.001
page	0.169	0.079	5301.5	2.129	=.033
trial	0.010	0.032	2190.4	0.327	
anomaly_type metric:layout poem	-0.138	0.088	3578.6	-1.576	
anomaly_type r&m:layout poem	0.124	0.056	6815.5	2.197	=.028
anomaly_type rhyme:layout poem	0.023	0.067	3918.2	0.343	
anomaly_type metric:MRRL_version inconsistent	0.033	0.073	2876.4	0.456	
anomaly_type r&m:MRRL_version inconsistent	0.107	0.052	5859.9	2.049	=.041
anomaly_type rhyme:MRRL_version inconsistent	-0.098	0.065	3429.6	-1.496	
layout poem:MRRL_version inconsistent	-0.064	0.035	5320.3	-1.828	=.068
wpos:page	0.185	0.066	7200.4	2.828	=.005
anomaly_type metric:layout poem:MRRL_version inconsistent	-0.154	0.073	2583.3	-2.100	=.036
anomaly_type r&m:layout poem:MRRL_version inconsistent	-0.012	0.052	6419.1	-0.235	
anomaly_type rhyme:layout poem:MRRL_version inconsistent	0.050	0.065	5027.6	0.776	

Note. The number of observation was 7458, the conditional R^2^ was 0.088 and marginal R^2^ was 0.008. Model Specification: log(lc_local + 1) ~ anomaly_type * layout * MRRL_version + wpos * page + trial + (anomaly_type + layout + MRRL_version | vp) + (1 | item)

### Discussion

The raw *load contribution* measure was introduced to account for selective re-reading.

*Re-reading of pre-rhyme.* We found that *rhyme* anomalies in both the *poem* and the *prose layout* induced re-reading of the pre-rhyme. Readers appear to utilize pre-rhymes for resolving the rhyme anomaly across lines.

For metrical anomalies it seems plausible that refixation of the pre-rhyme is not necessary for resolving metrical anomalies, when the expected rhyme scheme is met.

Strikingly, combined meter and rhyme anomalies (*r&m*) in the poem layout elicited the highest amount of re-reading of the pre-rhyme. The fact that rhyme-meter anomalies (*r&m*) only elicited re-reading in the *poem layout* suggests that, in prose layout, the combined absence of metrical, rhyme and visual layout cues appeared to leave readers disoriented, whereas readers could at least use the visual cues of strict poem layouts to check for the pre-rhyme in case of a severe anomaly. This, of course, requires that the anomalies had been noticed beforehand.

In general, the results indicate that readers were able to process the rhyme scheme, which they only could have accomplished by processing and representing phonetic and phonological information.

*Re-reading of local context.* For *metric* (and *r&m*) anomalies we expected more re-reading of the material preceding the anomaly in the current verse because it establishes the metrical pattern leading to the last word where the metric is broken. This hypothesis was supported for prose but not for poem layouts. This somewhat surprising interaction with layout may be due to the fact that re-reading the current verse in prose layout required jumping back to the previous line in some stimuli, whereas they could stay in the current line in poem layout. In our analysis, we operationalized local context as the six words preceding an anomaly. This window may have exceeded the number of words in the line though. Importantly, we found only rare re-visits of words farther than three words away. This suggests that readers remained within the current verse, and that words before it had no big impact on our results.

In summary, our findings on re-reading support the assumption that readers are sensitive to different types of rhythmic expectation violations. They react differently to metric than to rhyme anomalies, with vastly different re-fixation patterns.

## General Discussion

Our goal was to investigate silent MRRL reading. We hypothesized that readers would pick up the rhythmic patterns induced by the sound of words and their phonological stress pattern in metrically regular and rhymed language. We found a multitude of indicators for rhythmic subvocalization. Readers responded to metrical anomalies with longer reading times both on the anomalies themselves and by re-reading the preceding material. Similarly, rhyme, as well as *rhyme&meter* anomalies, were read longer and triggered systematic re-reading of pre-rhymes, particularly in the poem layout. This finding suggests that the metric and rhyme structure of the stanzas had been picked up very well. The differential effects for the anomaly types in different layouts, however, deserves some closer examination.

Increased fixation and reading times for *metric* anomalies were found for all four reading time measures, albeit only in the poem layout. Here, readers clearly seemed to be disrupted when the metrical grid was violated. The fact that readers appeared to stumble less over metrical anomalies in prose layouts is somewhat puzzling. Apparently, the overlay of line breaks and verse endings is crucial for establishing a verse’s metrical grid. This may have two possible reasons: Firstly, when lines are composed of two distinct verse segments, which in our stimuli was often the case in prose layout, they may have been processed as enjambments, as investigated by Koops van’t Jagt et al. (73), or simply as metrically disconnected. In the latter case, metrical processing may be impeded. This assumption is supported by the lack of metrical anomaly effects in our reading time data. In the former case, when the verse structure is experienced as intact even though it spans over two lines, the metrical structure should have been experienced as well. This assumption is corroborated by the finding of increased selective re-reading of the preceding words in prose layouts when regressive saccades were prompted by metric anomalies after first pass reading. These cases, albeit rare, suggest that metric anomalies in prose layouts do bear the potential to elicit specific eye movement responses.

Secondly, metrical anomalies may be more tolerable in prose layouts as long as the rhyme is intact. In the absence of visual poetic cues, the poetic form may just have been experienced as less strict.

*Rhyme* anomalies, on the other hand, caused stronger reading time effects in the *prose* layout in gaze durations and regression path durations. In the *poem* layout, *rhyme* anomalies show no such effect and appear to merely elicit an adoption of a different rhyme scheme, such as ABAC.

One explanation for the interaction with layout could be that, in the absence of a clear poetic visual form in *prose* layout, rhymes may serve as “poetic anchors” to support the structural expectations induced by the MRRL environment. In *poem* layout, visual cues and other stylistic devices may serve the same purpose, so that no such anchor is necessary. Therefore, rhyme violations become more tolerable, and readers easily accommodate rhyme deviations into a slightly diverging rhyme scheme.

This is in line with the notion that rhyme has the potential to end stanzas and poems as a rhythmic unit by eliciting a “sense of closure” (130), as a phenomenological experience. In the *poem* layout, a sense of closure can be induced by the visual ‘gestalt’ of a stanza/poem. The *function of closure* account is supported by Fechino et al. (36), who report overall longer first fixation durations, gaze durations and total reading times for rhyme words when presented in verse layout. They also report increased rereading probabilities for rhyme words in the prose layout, indicating that readers have processed the rhyme. In our consistent versions, however, we did not find this contrast.

Menninghaus and Wallot (96) reported longer total word reading times (total gaze durations) for poem with higher appreciation scores. At the same time, they found that rhyme anomalies had the strongest negative effect on appreciation, whereas metric anomalies reduced appreciation about half as much (but see 128 for a critique of the construct of “aesthetic emotions”). Could the reading time effects of anomalies and layout in our study hence be caused by differential effects of appreciation? The pattern of our results does not suggest so. Firstly, rhyme anomalies did not elicit stronger reading time increases than metric violations, it is rather the other way round. Secondly, both effects interacted with layout in different ways. Although we do not have data on how layout affects appreciation, we would suspect that the poem layout would receive higher appreciation scores than prose. If appreciation was the mediating variable, we would expect the shortest total word reading times for *rhyme* – and *r&m* – anomalies in *prose* layout, and the longest for *consistent* versions in *poem* layout, with *rhyme* anomalies in *poem* layouts, *metric* anomalies, and *consistent* version in *prose* layout somewhere in between. This is not what we found. Instead, our pattern of results suggest that readers’ reactions were due to specific processes of accommodation and repair caused by the very nature of the anomalies and the layout.

Scheepers et al. (119) found strong effects of rhyme anomalies in listeners’ pupillary responses, but the effects of metrical and other anomalies were much smaller. In our study we found the strongest effect on reading times for metrical anomalies. The two studies used very different dependent measures, as well as different stimuli types, and, most importantly, different presentation modalities. Spoken stimuli, as in Scheepers et al.’s study, provide all phonological cues necessary to directly perceive a rhythmic gestalt, whereas readers are forced to reconstruct the auditive gestalt from subvocalization of visual stimuli.

We assume that subvocalization is cognitively more demanding than listening to external speech, as subvocalization entails language production on top of visual language processing. If this is the case, the processing of rhymes spanning two lines may be particularly more demanding in silent reading than in listening. Metric processing however can be achieved via subvocalization of only a few local words and may therefore be less demanding, hence the different results. Moreover, pupillary responses in Scheepers et al.´s study probably reflect processes on an affective or aesthetic level, where rhymes or rhyme violations may trigger stronger responses.

Previous research states that readers generally adjust their reading style and pace to the text genre and that the poem layout is a relevant cue for such an adjustments (13; 51, 52; 96; 108; 148). In our study, the rhyme effects in gaze durations and RPDs suggest that MRRL is detectable without the layout cue. Hence, layout is neither necessary nor sufficient for identifying a MRRL-text as poetry. This is in line with conclusions drawn in Fechino et al’ (36, p. 13).

We also analyzed how different anomaly types triggered readers to re-read certain portions of text systematically. Re-reading time (load contribution) of the pre-rhyme revealed that readers captured the rhyme scheme in both layouts and used it for cross-line re-orientation towards the pre-rhyme when deviations from the overall dominant rhyme pattern in MRRL occurred. Interestingly, re-reading of pre-rhymes was activated strongest for rhyme-meter anomalies in layout poem, whereas no such effect was found for the prose layout.

Load contribution appear to be a sensitive measure in this specific case, since the results clearly suggest that readers did capture a combined rhyme and meter anomaly in poem layouts as an anomaly, thus experiencing a sense of violation of the overall metrical grid. However, the visual-spatial cues of a strict poem layout seem to somehow facilitate orientation towards the pre-rhyme. Taken together, the effects of RPD and LC suggest that the rhyme scheme had been picked up and processed by readers in both the poem and the prose layout. However, the visual cues of a poem layout made it easier to resolve severe rhyme and meter anomalies.

We also included two variables closely linked to pronunciation as indicators of subvocalization: *number of syllables* and the *consonant vowel quotient, cvq*, both residualized for word length (the *cvq* was also residualized for residual number of syllables, to keep both variables independent).

Previously, the *number of* s*yllables* in a word has been reported to have no additional effect on reading times beyond *word length* in normal reading (40 ). However, in our experiment on MRRL-reading, we found that all reading times were highly sensitive to syllables, on top of effects of word length and frequency, indicating that silent reading of MRRL is closely tied to pronunciation. In fact, SFDs only showed a clear syllable effect but no effect of word length, and thus appears to be linked more closely to pronunciation in our study. Crucially, the syllable effect was increased in text versions with anomalies in early processing measures (SFD and GAZE). This result in particular indicates that readers resorted to even more intense subvocalization when reading gets disturbed by MRRL anomalies, presumably narrowing the eye-(inner)-voice span even further.

Contrary to the assumption that a higher consonant density should reduce speakability and thus slow down subvocalization, the residual consonant vowel quotient did not affect reading times. We found that in our material, *cvq*s were highly correlated with residual syllable length (where word length had been cancelled out). Nevertheless, *cvq*s turned out to be a much better predictor for word skipping, where the number of syllables played virtually no role. Taken together, the findings suggest that fixation duration measures were strongly influenced by pronunciation *duration,* as approximated by syllable number, whereas skipping is more affected by *pronounceability* as approximated by the residual *cvq*.

At line beginnings, both *word-length* and word *category* strongly affected skipping. Line initial word skipping thus appears to depend upon both bottom-up perceptual features, such as shape, and top-down prediction-based information, such as word category. Presentation *layout,* however, did not have an additional effect. This result stands in contrast to the assumption that the poem layout itself induces more cautious reading at line beginnings (15). There was a notable difference between the materials of the two studies though: in the Blohm study, all lines in the poetry layout started with a capital letter (ibid. 72), whereas ours did not. Hence, capitalization might have attracted attention to line initial words, rather than a more cautious reading style.

We conclude that our finding indicates a high grade of synchronization of the eyes with inner speech (see Silva, Reis, Casaca, Petersson, & Faísca, 2016 for a discussion on the topic of voice-eye-lead), induced by the rhythmic structure of MRRL-language.

We would like to add some general remarks on subvocalization. There might be several levels of representing an “inner -rhythmic - voice” while reading MRRL silently: Ranging from an abstract representation of implicit prosody (compare 16) to the specific, conscious use of an inner voice (2). Also, automatized – yet vulnerable – vs. controlled rhythmic processing may play a role. However, for all ‘levels’, we presume that for rhythmic subvocalization of MRRL, phonological awareness (24; 92 ) is crucial for the detection of ‘the beats between and on the sounds’ (83; 134). Such a process, to us, may be closely related to the ability to induce a beat as well as to the potential to get entrained.

However, this first investigation of subvocalization in MRRL using eye-tracking measures can only shed initial light on the topic. Eye-movements may not bear a direct index of entrainment processes - but supposedly an additional one (compare 82; 101 ). That said, we would like to point out that other evidence for rhythm representation and processing comes from studies on musical notation reading, indicating a temporal, melodic and pause-bound representation of rhythm as reflected in eye movements (127).

Also, unlike other experiments which focused particularly on the interaction of metric anomalies and sentence processing (18, 19 ), our study had a somewhat broader starting point. The perception and processing of anomalies is just one factor among a variety of variables we looked at, among e.g., the layout variation and others. Most importantly, we were interested in MRRL induced reading styles *at other positions than the critical interest areas* to better understand how MRRL elicits rhythmic subvocalization and induces entrainment. Consequently, we had to use longer stimuli to be able to compare possible interactions. We also deliberately chose to not include comprehension or memory tasks (135; 148), because both tasks might have changed the reading style with respect to rhythmic subvocalization. We do presume that the processing of a poem’s rhythm can both guide and impede comprehension, however, this question was not within the scope of our study.

Another important aspect to discuss is the nature of the stimulus material itself. The poems were constructed in a way such that virtually no additional pausing, lengthening or shortening of syllables was required to realize a relatively uniform rhythm. The syllable structure thus aligned easily with the abstract metrical grid. We therefore think that a (linguistic) conceptual difference between meter and rhythm can be neglected in our study.

It also remains an open question, whether a reader’s subvocalization realizes the full rhythmic scope of MRRL or merely so in degrees. For example, if one is not experienced in expressive reciting, the prosody of ‘daily speech’ may shape subvocalization and comprise it in silent reading MRRL to a more binary stance, i.e., strong-weak stressing. Then, silent reading may be aligned solely with a ‘tactus’, i.e., the induced beat. Hence, subvocalization would not necessarily imply strong phrasal, intonational or modulating aspects.

However, given the assumption that this type of MRRL can produce a reasonably stable periodicity, i.e., ‘quasi-isochrony’ (105, 106 ), the following should hold: „all isochronous sequences are rhythmic, but not vice versa“ (112). That said, we propose that MRRL eliciting rhythmic subvocalization - which we found strong indicators for – may linger between idealized vs. empirical isochrony. In *silent* reading MRRL, related cognitive processes may not necessarily be provoked by the physical signal itself, but they are most certainly based on the „psychological tendency to superimpose an isochronous grid to a rhythmic sequence“ (112).

### Limitations

First, reading experiments using natural and, in particular, long texts are not only rare but also prone to error. Half of our stimuli, which were written specifically for the purpose of the experiment, included “anomalies” as described in the Design and Materials section. A rhyme-scheme error was spotted within the first stanza of “9 Leben”. Here, the ABAB-scheme was not met (“Morgen” in line 3, instead of “Lande”). However, we do believe that this did not carry weight for beat extraction and expectation of a rhythmic figure throughout the rest of the poem. Two minor corrections had to be made for two words in the stimuli during data collection. There was a mistake at the last word of stanza 5 (not a critical IA) in the modified version of item “Flüstern”, where the word „wertig“ had to be replaced with “fein”, as in the original version. This mistake was corrected from participant nr. 23 on. Another mistake was a small number of wrong apostrophe symbols and one orthographic mistake. This was corrected from participant nr. 29 on. For data analysis, the affected data points on the two IAs were coded accordingly and did not affect the overall results.

Secondly, one can criticize that the anomalies chosen have not been consistent with respect to the *type* of rhythmic deviation, e.g., adding one or two syllables could have elicited floating stress, diffraction, or even one more “beat”. Hence, we cannot distinguish which specific kind of rhythmic discrepancy led to which specific eye-movement reaction. It might be a challenging task to integrate such uniformity into these kinds of long (poetic) text stimuli, particularly because the rhythm of each traditionally composed poem is individual and is therefore describable only in comparison with a projected meter (see Burdorf, 1997, p. 69-73 for a discussion of rhythm in poetry).

Third, since each type of anomaly always occurred at the same position, effects of anomaly type might be confounded with other position-related variables, such as practice effects. This possibility needs to be addressed in future research, which is why another experimental design has been set up including a rotation system of manipulations. It will be published elsewhere. However, since the MRRL-text differed only slightly across different conditions, we believe that it might be very demanding to mentally represent and update the beat or rhythmic structure in the poem (or prose) layout while, at the same time, remembering where exactly which rhythmic deviation had occurred.

Fourth, a potential weakness of the analysis is that other lexical variables of successor words were not included. Word length and frequency, for instance, could affect parafoveal word recognition and saccade planning. However, since we were mainly interested in pronunciation-related parafoveal processing, other variables were not additionally analyzed. Also, a variable ‘position within line’ and ‘stanza’ could have been integrated to analyze where, within a stanza, reading might change, especially since the stanza structure repeats periodically. This should be considered in replications of the study.

### Conclusion

We found several indicators of rhythmical subvocalization in silent MRRL reading. Metrical anomalies elicited longer single fixation durations, gaze durations, and regression path durations, while rhyme anomalies triggered re-reading of the rhyme-primes. If readers had not picked up the rhythmical structure from the stimuli, our meter and rhyme manipulations would have gone unnoticed and no difference in reading for the consistent and inconsistent versions should have been measured. The clear anomaly effects thus strongly suggest rhythmic subvocalization in silent MRRL reading.

Strict verse-by-verse poem layout can strengthen rhythmic expectations, leading to robust meter anomaly effects. MRRL in prose layout, on the other hand, elicited rhyme effects, indicating that the language was also processed rhythmically. However, due to the lack of visual formal cues, rhymes were utilized as important anchor points to establish the poetic gestalt.

With respect to general parameters indicating rhythmic subvocalization in silent reading, we argued that effects of pronounceability, such as a high correlation of fixation duration measures with the pronunciation length of words, as measured by the number of syllables, clearly speak in favor of a close alignment of eye-movements and the inner voice. Moreover, we found evidence for parafoveal processing of features related to pronounceability, further supporting the subvocalization assumption. Future research will have to show whether this finding extends to other variables related to pronunciation such as neighborhood density or sonority (149, 148).

Our results suggest that rhythmicity in subvocalization may be graded depending on how easy or difficult it is to pick up an MRRL sound "gestalt". Thus, the alignment of the eyes with the inner voice may also be graded. Future research will have to investigate the extent to which rhythmic subvocalization is evoked in non-MRRL type poems. It is possible that an inherent beat is needed for these types of effects, but this remains an open question for future research.

### Ethics and Conflict of Interest

The authors declare that the contents of the article are in agreement with the ethics described in http://biblio.unibe.ch/portale/elibrary/BOP/jemr/ethics.html and that there is no conflict of interest regarding the publication of this paper.

### Acknowledgements

We wish to thank Dennis Erath for his support in running the experiment, and Evelyn Ferstl, Simon Kirsch, Kyla McConnell and Lisa Zacharski for fruitful discussions and comments. The article processing charge was funded by the Baden-Wuerttemberg Ministry of Science, Research and Art and the University of Freiburg in the funding programme Open Access Publishing.
