# Supplementary material for: Rhythmic subvocalization: An eye-tracking study on silent poetry reading
Source: J Eye Mov Res. 2021 Sep 14;13(3):10.16910/jemr.13.3.5. doi: 10.16910/jemr.13.3.5 (PMC8557949; doi:10.16910/jemr.13.3.5)
Supplement: Supplementary file 1 [file jemr-13-03-e-appendix-01.pdf]

Prose Layout

9 Leben

Auf blauem Gebirge am Rande da liegt still
ein Walde allein und birgt im hebenden Morgen mit
nebligem Dampfen den Stein. Er atmete kalt all
die Winde und seufzte mitunter ganz leis – zu
bergen ist schließlich ein Kinde. Er mühet sich
redlich mit Fleiß. 1 | **Pflicht**. 2

Am helligsten Tage im Jahre es schnell
eingepflanzet im wurd, drauf folgte die
Wachstumsfanfare, die Äste war'n bald
festgezurrt. Es schüttelte manchmal die Zapfen
und surrte dazwischen mal rasch, die Wurzeln
gediehen zu Stapfen, kaum eine war jemals je lasch. 1 | **entkräftet**. 4

Getreulich gedieh es zum Baume. Es stärkte
das Wasser den Stamm. Es hob seine Arme gen
Raume und stand nun wie andere stramm.
Er rüttelte heftig die Nadeln und achtete
nicht der Gefahr, die Urgründe hatten zu
tadeln und zeugen von sich planetar. 1 | **universell**. 4

Doch oft vergisst er die Winter und steht
dann dort wie allein. Im Wald ohne Bäume
dahinter 1 | **hintendran** 2 fehlt ihm die Verwandtschaft zu sein. 1 | **zum reifen**. 4

Poem Layout

9 Leben

Auf blauem Gebirge am Rande
da liegt still ein Walde allein
und birgt im hebenden Morgen
mit nebligem Dampfen den Stein

Er atmete kalt all die Winde
und seufzte mitunter ganz leis –
zu bergen ist schließlich ein Kinde
Er mühet sich redlich mit Fleiß 1 | **Pflicht** 2

Am helligsten Tage im Jahre
es schnell eingepflanzet im wurd
drauf folgte die Wachstumsfanfare
die Äste war'n bald festgezurrt

Es schüttelte manchmal die Zapfen
und surrte dazwischen mal rasch
die Wurzeln gediehen zu Stapfen
kaum eine war jemals je lasch 1 | **entkräftet** 2

Getreulich gedieh es zum Baume
Es stärkte das Wasser den Stamm
Es hob seine Arme gen Raume
und stand nun wie andere stramm

Er rüttelte heftig die Nadeln
und achtete nicht der Gefahr
die Urgründe hatten zu tadeln
und zeugen von sich planetar 1 | **universell** 4

Doch oft vergisst er die Winter
und steht dann dort wie allein
im Wald ohne Bäume dahinter 1 | **hintendran** 2
fehlt ihm die Verwandtschaft zu sein 1 | **zum reifen** 4

Numbers indicate:

(1) original text,
(2) substitution of rhyme with original number of syllables
(3) change of prominent metrical figure by adding one or two syllables with rhyme being maintained
(4) change of prominent metrical figure by adding or subtracting one or two syllables, with substitution of rhyme
Note that only text corpus, thus no titles, were presented to readers.

Prose Layout

Dancing Queen

Schon in meiner Jugend Flüge ging ich gerne
nächtens fort, und im Taumel vieler Krüge
sucht’ ich auf so manchen Ort. Ja da waren
bunte Lichter und Bewegung in dem Fuß,
laute Stunden wurden dichter und im
Feuertopf schon Ruß. $_1$ | **Dreck**. $_2$

Denn da war ein fröhlich’ Feiern bis die
Nachbarn fandens öd, mancher musste mal
auch reiern, wenn’s der Stirne wurd’ zu blöd.
Falls die Meute quirlig wurde, gab’s
tags drauf ein Hoch darauf und der Quatsch ward
noch absurde mit der Kasperkappe auf. $_1$ | **obenauf**. $_3$

Morgen wurd’s und wieder Abend, immer
schlimmer trieb’s das Bein… Muskeln
sich in Musik laben und dazwischen
glücklich sein. Später, früher, ohne Uhren,
drehten Zeiger ihren Kreis, folgten froh den
Notenspuren auf dem Weg zum Chorusgleis. $_1$ | **Chorusplatze**. $_4$

So verging so manche Runde, bis die Platte
nicht mehr lief kehlten Lieder noch
die Kunde $_1$ | **Info** $_2$ - bis der Guard zum Gehen rief. $_1$ | **befahl** $_4$

Poem Layout

Dancing Queen

Schon in meiner Jugend Flüge
ging ich gerne nächtens fort,
und im Taumel vieler Krüge
sucht’ ich auf so manchen Ort.

Ja da waren bunte Lichter
und Bewegung in dem Fuß,
laute Stunden wurden dichter
und im Feuertopf schon Ruß. $_1$ | **Dreck**. $_2$

Denn da war ein fröhlich’ Feiern
bis die Nachbarn fandens öd,
mancher musste mal auch reiern,
wenn’s der Stirne wurd’ zu blöd.

Falls die Meute quirlig wurde,
gab’s tags drauf ein Hoch darauf
und der Quatsch ward noch absurde
mit der Kasperkappe auf. $_1$ | **obenauf**. $_3$

Morgen wurd’s und wieder Abend,
immer schlimmer trieb’s das Bein…
Muskeln sich in Musik laben
und dazwischen glücklich sein.

Später, früher, ohne Uhren,
drehten Zeiger ihren Kreis,
folgten froh den Notenspuren
auf dem Weg zum Chorusgleis. $_1$ | **Chorusplatze**. $_4$

So verging so manche Runde,
bis die Platte nicht mehr lief
kehlten Lieder noch die Kunde $_1$ | **Info** $_2$
- bis der Guard zum Gehen rief. $_1$ | **befahl** $_4$

Numbers indicate:

(1) original text,
(2) substitution of rhyme with original number of syllables
(3) change of prominent metrical figure by adding one or two syllables with rhyme being maintained
(4) change of prominent metrical figure by adding or subtracting one or two syllables, with substitution of rhyme
Note that only text corpus, thus no titles, were presented to readers.

Prose Layout

Flüstern

Es blinken einen schön die Sterne wie Glitzerstuck am Himmel an, als sängen sie uns ihre Ferne und leuchten uns der Nächte Bahn. Es sonnt sich tags das Prachtgestirn, um dann im Dunkel bleich zu leuchten, die Wolken wirbeln feinsten Zwirn, bis sie die Wipfel taubefeuchten. 1 | **taubeflecken**. 2

Es wallen drunten weiche Wellen wie Dünen aus verwunsch'nem Naß und drauf da hüpfen Möwen Dellen und picken Fisch' ohn Unterlaß. Es türmt am Lande sich der Berg in Grau und Weiß da steht er stramm und hinter ihm ein Hügelzwerg,
sie bilden beide einen Kamm. 1 | **Gebirgskamm**. 3

Es wächst der Saum der kühlen Wälder als Spitzen grün ins Blau hinein, bald weiter strecken gelb sich Felder und bilden Ähren stark und fein. Auch blüht darin so manch ein Kind zu einer Blume auf und färbet die Umwelt bunt und wirkt geschwind, bis dass das Alter es beerbet. 1 | **übertraget**. 4

So zieht seit Jahren der Planet mit allen von uns seine Bahnen und sorgt, dass stets der Wind drauf weht 1 | **bläst** 2 der Zeiten Atem von den Ahnen. 1 | **Altvordern**. 4

Poem Layout

Flüstern

Es blinken einen schön die Sterne
wie Glitzerstuck am Himmel an
als sängen sie uns ihre Ferne
und leuchten uns der Nächte Bahn

Es sonnt sich tags das Prachtgestirn
um dann im Dunkel bleich zu leuchten
die Wolken wirbeln feinsten Zwirn
bis sie die Wipfel taubefeuchten 1 | **taubeflecken** 2

Es wallen drunten weiche Wellen
wie Dünen aus verwunsch'nem Naß
und drauf da hüpfen Möwen Dellen
und picken Fisch' ohn Unterlaß

Es türmt am Lande sich der Berg
in Grau und Weiß da steht er stramm
und hinter ihm ein Hügelzwerg
sie bilden beide einen Kamm 1 | **Gebirgskamm** 3

Es wächst der Saum der kühlen Wälder
als Spitzen grün ins Blau hinein
bald weiter strecken gelb sich Felder
und bilden Ähren stark und fein

Auch blüht darin so manch ein Kind
zu einer Blume auf und färbet
die Umwelt bunt und wirkt geschwind
bis dass das Alter es beerbet 1 | **übertraget** 4

So zieht seit Jahren der Planet
mit allen von uns seine Bahnen
und sorgt, dass stets der Wind drauf weht 1 | **bläst** 2
der Zeiten Atem von den Ahnen 1 | **Altvordern** 4

Numbers indicate:

(1) original text,
(2) substitution of rhyme with original number of syllables
(3) change of prominent metrical figure by adding one or two syllables with rhyme being maintained
(4) change of prominent metrical figure by adding or subtracting one or two syllables, with substitution of rhyme
Note that only text corpus, thus no titles, were presented to readers.

Prose Layout

Glühwürmchen

Wir hatten keine Kerzen bei und auch die Taschenlampen nicht, es war ja auch ganz einerlei, wir liefen gut auf freie Sicht. Die Taschen waren voll bepackt mit allem was die Welt begehrt, wir gingen Gleichschritt, fast im Takt, doch schnell war's meiste aufgezehrt. $_{1}$ | **aufgebraucht.** $_{2}$

Der Hunger dennoch war vorbei, was andres war viel lockender… Bald Nacht vom Tage ganz entzwei und Füße trabten stockender. Die Bäume jäh schon trennten sich und vor uns liegend Wassers Gang. Das Ufer drüben nur ein Strich, er ging am untern Himmel lang. $_{1}$ | **entlang.** $_{3}$

Da stoppten wir mit Atmung still und blickten in die Weite hin und hörten kurz noch Grillen schrill, doch zügig war'n sie aus dem Sinn. Denn lichternd war's am dunklen See und wieder waren alle da. So manches knipste schnell noch Klee hell blinkend an, wir machten Ah! $_{1}$ | **Ohoh**! $_{4}$

Die Würmchen flogen froh im Schwarm und unsre Augen hintendrein, beim bloßen Schauen wurd es warm. $_{1}$ | **heiß**. $_{2}$ So darf im Mai das Glühen sein. $_{1}$ | **werden**. $_{4}$

Poem Layout

Glühwürmchen

Wir hatten keine Kerzen bei
und auch die Taschenlampen nicht
es war ja auch ganz einerlei
wir liefen gut auf freie Sicht

Die Taschen waren voll bepackt
mit allem was die Welt begehrt
wir gingen Gleichschritt, fast im Takt
doch schnell war's meiste aufgezehrt $_{1}$ | **aufgebraucht** $_{2}$

Der Hunger dennoch war vorbei
was andres war viel lockender…
Bald Nacht vom Tage ganz entzwei
und Füße trabten stockender

Die Bäume jäh schon trennten sich
und vor uns liegend Wassers Gang
Das Ufer drüben nur ein Strich
er ging am untern Himmel lang $_{1}$ | **entlang** $_{3}$

Da stoppten wir mit Atmung still
und blickten in die Weite hin
Und hörten kurz noch Grillen schrill
doch zügig war'n sie aus dem Sinn

Denn lichternd war's am dunklen See
und wieder waren alle da
So manches knipste schnell noch Klee
hell blinkend an, wir machten Ah! $_{1}$ | **Ohoh**! $_{4}$

Die Würmchen flogen froh im Schwarm
und unsre Augen hintendrein
beim bloßen Schauen wurd es warm $_{1}$ | **heiß** $_{2}$
So darf im Mai das Glühen sein $_{1}$ | **werden** $_{4}$

Numbers indicate:

(1) original text,
(2) substitution of rhyme with original number of syllables
(3) change of prominent metrical figure by adding one or two syllables with rhyme being maintained
(4) change of prominent metrical figure by adding or subtracting one or two syllables, with substitution of rhyme
Note that only text corpus, thus no titles, were presented to readers.

Prose Layout

Im Hüteland

Ein Heben wird's im Hüteland und sicher freut's die Welt zum Gruß. So mancher weilet kurz im Stand, froh weiter geht es, stets zu Fuß. Die Frösche quaken spät noch laut und Weiher laden ein zum Bad. Wer sich unter die Grünsten traut dem wurd die Stimmung nimmer fad. 1 | **öd.** 2

Ein Tollen tönt am Waldesrand, wo sich das Laub im Winde bäumt und Knollen wachsen aus dem Sand, der sich hier klumpig erdig bräunt. Die Hasen hüpfen querfeldein, sie reißen manche Blume um. Die Rehe sonntags hintendrein und winters geht es andersrum. 1 | **andersherum.** 3

Am zweiten Tümpel sitzt der Bär, die Tatze tastet Fischefang. Das Fell ist von den Fliegen schwer und plätschernd blubbern Blasen bang. Der Apfelbaum steht stramm noch da, dran auch die alte Schaukel hängt. Drauf sitz ein Rabe und macht Krah bis es den Wurm zur Kehle drängt. 1 | **hinzieht.** 4

Im Sommerreigen einen Gang, den tut man auch noch heute gern. Der Städter sucht vergebens lang 1 | **hier** 2 Natur in dieser Form stets fern. 1 | **auswärts.** 4

Poem Layout

Im Hüteland

Ein Heben wird's im Hüteland
und sicher freut's die Welt zum Gruß
So mancher weilet kurz im Stand
froh weiter geht es, stets zu Fuß

Die Frösche quaken spät noch laut
und Weiher laden ein zum Bad
Wer sich unter die Grünsten traut
dem wurd die Stimmung nimmer fad 1 | **öd** 2

Ein Tollen tönt am Waldesrand
wo sich das Laub im Winde bäumt
und Knollen wachsen aus dem Sand
der sich hier klumpig erdig bräunt

Die Hasen hüpfen querfeldein
sie reißen manche Blume um
Die Rehe sonntags hintendrein
und winters geht es andersrum 1 | **andersherum** 3

Am zweiten Tümpel sitzt der Bär
die Tatze tastet Fischefang
Das Fell ist von den Fliegen schwer
und plätschernd blubbern Blasen bang

Der Apfelbaum steht stramm noch da
dran auch die alte Schaukel hängt
Drauf sitz ein Rabe und macht Krah
bis es den Wurm zur Kehle drängt 1 | **hinzieht** 4

Im Sommerreigen einen Gang
den tut man auch noch heute gern
Der Städter sucht vergebens lang 1 | **hier** 2
Natur in dieser Form stets fern 1 | **auswärts** 4

Numbers indicate:

(1) original text,
(2) substitution of rhyme with original number of syllables
(3) change of prominent metrical figure by adding one or two syllables with rhyme being maintained
(4) change of prominent metrical figure by adding or subtracting one or two syllables, with substitution of rhyme
Note that only text corpus, thus no titles, were presented to readers.

Prose Layout

Klimawandel

Dort unten in dem Tale saß ich auf
leichten Wiesen und sah im Himmelssaale
die Wölklein fleißig spießen. Sah zu
der blanken Sonne, es war mir wie ein
Traum, sie bahnte sich mit Wonne in
einen Tannensaum. $_{1}$ | **Tannenwald.** $_{2}$

Die Helle war wie bebend in
Freudesmelodie durch alle Blüten
webend sang sie voll Harmonie. Du
bist zur besten Stunde o Läufermensch,
hier grad, du bist's für den die Runde
hier dringt zur Waldesnaht. $_{1}$ | **Waldesziernaht.** $_{3}$

Du weißt wie es wird werden, wenn ich
gewandert bin, der Nächte Zapfensterben
ein Wandel ohne Sinn. Noch sitzt du in
dem Lande, das ich geschaffen hab zum
Schutze nicht imstande, bereitest mir
ein Grab. $_{1}$ | **Ende.** $_{4}$

Die Strahlen sah ich fallen, mir wurd's
im Herzen schwer, ein „Bitte!“ wollt' ich
lallen, $_{1}$ | **nuscheln,** $_{2}$ da ging ihr Rad nicht mehr. $_{1}$ | **weiter.** $_{4}$

Poem Layout

Klimawandel

Dort unten in dem Tale
saß ich auf leichten Wiesen
und sah im Himmelssaale
die Wölklein fleißig spießen

Sah zu der blanken Sonne
es war mir wie ein Traum
sie bahnte sich mit Wonne
in einen Tannensaum $_{1}$ | **Tannenwald** $_{2}$

Die Helle war wie bebend
in Freudesmelodie
durch alle Blüten webend
sang sie voll Harmonie

Du bist zur besten Stunde
o Läufermensch, hier grad
du bist's für den die Runde
hier dringt zur Waldesnaht $_{1}$ | **Waldesziernaht** $_{3}$

Du weißt wie es wird werden
wenn ich gewandert bin
der Nächte Zapfensterben
ein Wandel ohne Sinn

Noch sitzt du in dem Lande
das ich geschaffen hab
zum Schutze nicht imstande
bereitest mir ein Grab $_{1}$ | **Ende** $_{4}$

Die Strahlen sah ich fallen
mir wurd's im Herzen schwer
ein „Bitte!“ wollt' ich lallen $_{1}$ | **nuscheln** $_{2}$
da ging ihr Rad nicht mehr $_{1}$ | **weiter** $_{4}$

Numbers indicate:

(1) original text,
(2) substitution of rhyme with original number of syllables
(3) change of prominent metrical figure by adding one or two syllables with rhyme being maintained
(4) change of prominent metrical figure by adding or subtracting one or two syllables, with substitution of rhyme
Note that only text corpus, thus no titles, were presented to readers.

Prose Layout

Normal

Noch ist die Stunde schwitzend still und Töne sind kaum mehr zu hören. Doch wer dem Zirpen lauschen will, lässt sich von keinem Klappern stören. Hier ist die Freude an dem Kleinen, dem Lächeln, das dir jemand schenkt. Und hörst Du mal ein Kleinkind weinen, bestaune wie die Stimme lenkt. 1 | **führt.** 2

Verfrüht das Wort im Angesicht von all dem guten Wünschemeinen. Es glüht am Himmel Sommerslicht und auf den Körpern trägt man Leinen. Es wird die Wärme stets begleiten der Sonnenstrahl zu kühlem Ort, bis Vögel unser Wissen leiten, bald weht die Kühle Hitze fort. 1 | **weithinfort.** 3

Das Rascheln in den grünen Blättern verkündet nächstens kalten Hauch, stets ahnend schon das Blitzewettern verkünden Schwalben solches auch. Erheitern lässt sich dann der Geist, im Dulden ist die Kraft gegeben. Wer darum schon im Stillen weiß, kann solche Zeiten gut durchleben. 1| **überstehen.** 4

So wirkt seit Alters her die Erde. Ob Winter, Frühling, Sommer, Herbst. Was Schwüle ist auch Frost mal werde 1 | **wird** 4 und jedes Sein sein Gegen erbt. 1 | **empfängt.** 4

Poem Layout

Noch ist die Stunde schwitzend still
und Töne sind kaum mehr zu hören
Doch wer dem Zirpen lauschen will
lässt sich von keinem Klappern stören

Hier ist die Freude an dem Kleinen
dem Lächeln, das dir jemand schenkt
Und hörst Du mal ein Kleinkind weinen
bestaune wie die Stimme lenkt 1 | **führt** 2

Verfrüht das Wort im Angesicht
von all dem guten Wünschemeinen
Es glüht am Himmel Sommerslicht
und auf den Körpern trägt man Leinen

Es wird die Wärme stets begleiten
der Sonnenstrahl zu kühlem Ort
bis Vögel unser Wissen leiten
bald weht die Kühle Hitze fort 1 | **weithinfort** 3

Das Rascheln in den grünen Blättern
verkündet nächstens kalten Hauch
stets ahnend schon das Blitzewettern
verkünden Schwalben solches auch

Erheitern lässt sich dann der Geist
im Dulden ist die Kraft gegeben
Wer darum schon im Stillen weiß
kann solche Zeiten gut durchleben 1| **überstehen** 4

So wirkt seit Alters her die Erde
Ob Winter, Frühling, Sommer, Herbst
Was Schwüle ist auch Frost mal werde 1 | **wird** 4
und jedes Sein sein Gegen erbt 1 | **empfängt** 4

Normal

Numbers indicate:

(1) original text,
(2) substitution of rhyme with original number of syllables
(3) change of prominent metrical figure by adding one or two syllables with rhyme being maintained
(4) change of prominent metrical figure by adding or subtracting one or two syllables, with substitution of rhyme
Note that only text corpus, thus no titles, were presented to readers.
